# The effect of lanthanum on growth and gene expression in a facultative methanotroph

**DOI:** 10.1111/1462-2920.15685

**Published:** 2021-08-12

**Authors:** Andrew T. Crombie

**Affiliations:** ^1^ School of Biological Science University of East Anglia Norwich NR4 7TJ UK; ^2^ School of Environmental Science University of East Anglia Norwich NR4 7TJ UK

## Abstract

The biological importance of lanthanides has only recently been identified, initially as the active site metal of the alternative methanol dehydrogenase (MDH) Xox‐MDH. So far, the effect of lanthanide (Ln) has only been studied in relatively few organisms. This work investigated the effects of Ln on gene transcription and protein expression in the facultative methanotroph *Methylocella silvestris* BL2, a widely distributed methane‐oxidizing bacterium with the unique ability to grow not just on methane but also on other typical components of natural gas, ethane and propane. Expression of calcium‐ or Ln‐dependent MDH was controlled by Ln (the lanthanide switch) during growth on one‐, two‐ or three‐carbon substrates, and Ln imparted a considerable advantage during growth on propane, a novel result extending the importance of Ln to consumers of this component of natural gas. Two Xox‐MDHs were expressed and regulated by Ln in *M. silvestris*, but interestingly Ln repressed rather than induced expression of the second Xox‐MDH. Despite the metabolic versatility of *M. silvestris*, no other alcohol dehydrogenases were expressed, and in double‐mutant strains lacking genes encoding both Ca‐ and Ln‐dependent MDHs (*mxaF* and *xoxF5* or *xoxF1)*, growth on methanol and ethanol appeared to be enabled by expression of the soluble methane monooxygenase.

## Introduction

Methane, the most abundant hydrocarbon in the atmosphere and a potent greenhouse gas, is one of the most significant contributors to climate change (Saunois *et al*., 2016). Biogenic methane results from the metabolism of methanogenic archaea, but in addition, thermogenic methane‐rich natural gas is produced as a result of heat‐ and pressure‐mediated breakdown of buried organic material. Much natural gas is released to the atmosphere as a result of fossil fuel extraction, but naturally released thermogenic methane, from diffuse seepage or point releases such as macro‐seeps, mud volcanoes and volcanoes, accounts for 14% of all methane from natural sources and usually contains other short‐chain alkanes, mostly ethane and propane. Approximately 3 and 2 Tg y^−1^ of ethane and propane, respectively, are released to the atmosphere in natural gas (Etiope and Ciccioli, 2009; Dalsøren *et al*., 2018), which sometimes contains up to 35 vol% ethane and propane (Etiope and Klusman, 2010; Schimmelmann *et al*., 2018). Release of methane to the atmosphere is reduced by methane‐oxidizing microbes (methanotrophs), which consume over half of the methane produced in soils, sediments and the water column (Reeburgh, 2007) and emissions of ethane and propane are similarly mediated by microbes, but whose relative contribution is unknown (Beilen and Funhoff, 2005; Shennan, 2006; Kinnaman *et al*., 2007; Redmond *et al*., 2010).

The genus *Methylocella* comprises several species of aerobic facultative methanotrophs from the Alphaproteobacteria (type II), which are active in many environments and are sometimes the most abundant methanotrophs (Farhan Ul Haque *et al*., 2020). *Methylocella* is unique among methanotrophs as it grows robustly on a range of multi‐carbon compounds, including, for several of the *Methylocella* strains, not only methane but also ethane and propane (Dedysh *et al*., 2005; Crombie and Murrell, 2014; Farhan Ul Haque *et al*., 2019). These short‐chain alkanes are oxidized by soluble diiron centre monooxygenases to the corresponding alcohols: methanol, ethanol and 1‐ and 2‐propanol (Crombie and Murrell, 2014).

Methanol dehydrogenase (MDH) is the second enzyme of the methane metabolic pathway, catalyzing the conversion of methanol to formaldehyde. For many years, this was thought to be exclusively carried out, in methanotrophs, by a soluble pyrroloquinoline quinone (PQQ)‐containing periplasmic enzyme (Mxa‐MDH), made up of large and small subunits (MxaF and MxaI), in an α_2_β_2_ structure, with a calcium ion at the active site (Anthony, 2004). A specific cytochrome *c*_L_ (MxaG) accepts electrons from MDH, and another protein, MxaJ, annotated as a solute‐binding protein but of unknown function, is required for functional MDH (Amaratunga *et al*., 1997), and several genes at the same locus are involved in metal insertion and enzyme assembly and maturation. Recently, it was shown that a gene often present in methylotrophs (*xoxF*) encodes an homologous MDH (Xox‐MDH, approximately 50% amino acid identity between XoxF and MxaF), which uses a rare earth lanthanide (Ln), rather than calcium as cofactor (Fitriyanto *et al*., 2011; Hibi *et al*., 2011; Nakagawa *et al*., 2012; Pol *et al*., 2014), reviewed by Picone and Op den Camp (2018). The *xox* gene clusters also typically encode MxaG and MxaJ homologues (XoxG and XoxJ), but Xox‐MDH does not usually include a small subunit (although the enzyme from *Candidatus* Methylomirabilis oxyfera was purified as an α_2_β_2_ hybrid of XoxF and MxaI; Wu *et al*., 2015). Several studies have suggested that Xox‐MDH is as environmentally relevant, if not more so, than Mxa‐MDH (Taubert *et al*., 2015; Chistoserdova, 2016; Huang *et al*., 2019; Li *et al*., 2019), and it is also worth noting that rare earth elements are not, in fact, particularly rare in the Earth's crust (Tyler, 2004). Knowledge of Ln‐dependent MDH has enabled identification of Ln‐dependent methanol utilization in methanotrophs, methylotrophs, and members of genera not previously known for methylotrophy (Pol *et al*., 2014; Lv *et al*., 2018; Farhan Ul Haque *et al*., 2019; Huang *et al*., 2019; Kato *et al*., 2020). Based on sequence data, five clades of Xox‐MDHs can be identified, XoxF1–XoxF5, with additional Ln‐dependent homologues with activity towards other substrates (Keltjens *et al*., 2014). Clade 5 appears to be the most abundant, although many organisms contain multiple enzymes from the same or different clades (Taubert *et al*., 2015). Several Xox enzymes have been purified and the structures of examples from clades 2 and 5 have been determined, in addition to Mxa‐MDH. In both Mxa‐MDH and Xox‐MDH, the large subunit forms a superbarrel made up of eight four‐stranded beta sheets, arranged radially like the blades of a propeller with, in Mxa‐MDH, the small subunit folded round the surface (Anthony, 2004; Keltjens *et al*., 2014; Pol *et al*., 2014; Deng *et al*., 2018; Jahn *et al*., 2018; Good *et al*., 2020).

Both Mxa‐ and Xox‐MDHs contain a conserved catalytic Asp residue (position D303 in MxaF from *Methylorubrum extorquens*, formerly *Methylobacterium extorquens*) and the Ln‐dependent Xox‐MDHs can be identified by a second Asp residue two positions downstream, replacing Ala in MxaF (A305 in MxaF from *M. extorquens*), required for coordination of the lanthanide atom (Anthony, 2004; Keltjens *et al*., 2014; Good *et al*., 2020).

The advantage to a methanotroph of this methanol‐oxidizing flexibility is unclear, but it may be important as a response to changing environmental conditions or for controlling the symbiotic transfer of metabolites (e.g. methanol) to other members of the microbial community (Krause *et al*., 2016; Yu *et al*., 2017).

Although several examples lack one or other form of MDH, both Mxa‐MDH and Xox‐MDH are present in many methylotrophs and methanotrophs, where they are differentially regulated by the lanthanides also required for Xox‐MDH activity (Skovran and Martinez‐Gomez, 2015; Chistoserdova, 2019). This ‘lanthanide switch’ has been studied in a few methylotrophs and methanotrophs (Keltjens *et al*., 2014; Skovran and Martinez‐Gomez, 2015; Chistoserdova, 2016; Semrau *et al*., 2018; Chistoserdova, 2019; Skovran *et al*., 2019), demonstrating that in the presence of Ln, transcription and expression of Mxa‐MDH is repressed and that of Xox‐MDH is enhanced. Our understanding of the mechanisms of regulation of MDH, both in response to different substrates and to metals, is incomplete, but the best‐studied system is probably from *Methylorubrum extorquens*. The genome of this methylotroph encodes a pair of two‐component regulators, *mxcQE* and *mxbDM*, and a response regulator *mxaB*, all of which are proposed to be involved in Ln‐dependent regulation of methanol oxidation, and in addition the XoxF protein itself was shown to be part of the regulatory cascade (reviewed by Skovran *et al*., 2019 and Chistoserdova, 2019). The lanthanide switch has been investigated in a few methanotrophs including the alphaproteobacterium *Methylosinus trichosporium* OB3b, and the gammaproteobacteria *Methylomonas* sp. strain LW13 and *Methylotuvimicrobium buryatense* 5GB1C (formerly *Methylomicrobium*) (Farhan Ul Haque *et al*., 2015; Chu *et al*., 2016; Chu and Lidstrom, 2016; Zheng *et al*., 2018). Clearly, there are differences in the regulation of the lanthanide switch between facultative methylotrophs (*Methylorubrum*) and many methanotrophs. For example, neither *Methylosinus trichosporium* OB3b nor *Methylotuvimicrobium buryatense* 5GB1C contain close homologues of *mxcQE* or *mxbDM*, although both contain sensor histidine kinases which may sense lanthanides (Semrau *et al*., 2018; Skovran *et al*., 2019).

The role of lanthanides in biology was only recently identified (Cotruvo, 2019) and much remains unknown or poorly understood regarding the importance and role of these elements in one‐ and multi‐carbon metabolism. The genus *Methylocella* contains facultative methanotrophs able to grow well on both one‐carbon and multi‐carbon compounds and the aims of this study were to take the first steps towards an understanding of the role of lanthanides in the best‐studied example, *Methylocella silvestris* BL2.

## Results and discussion

### MDH gene transcription

**Figure 1:**
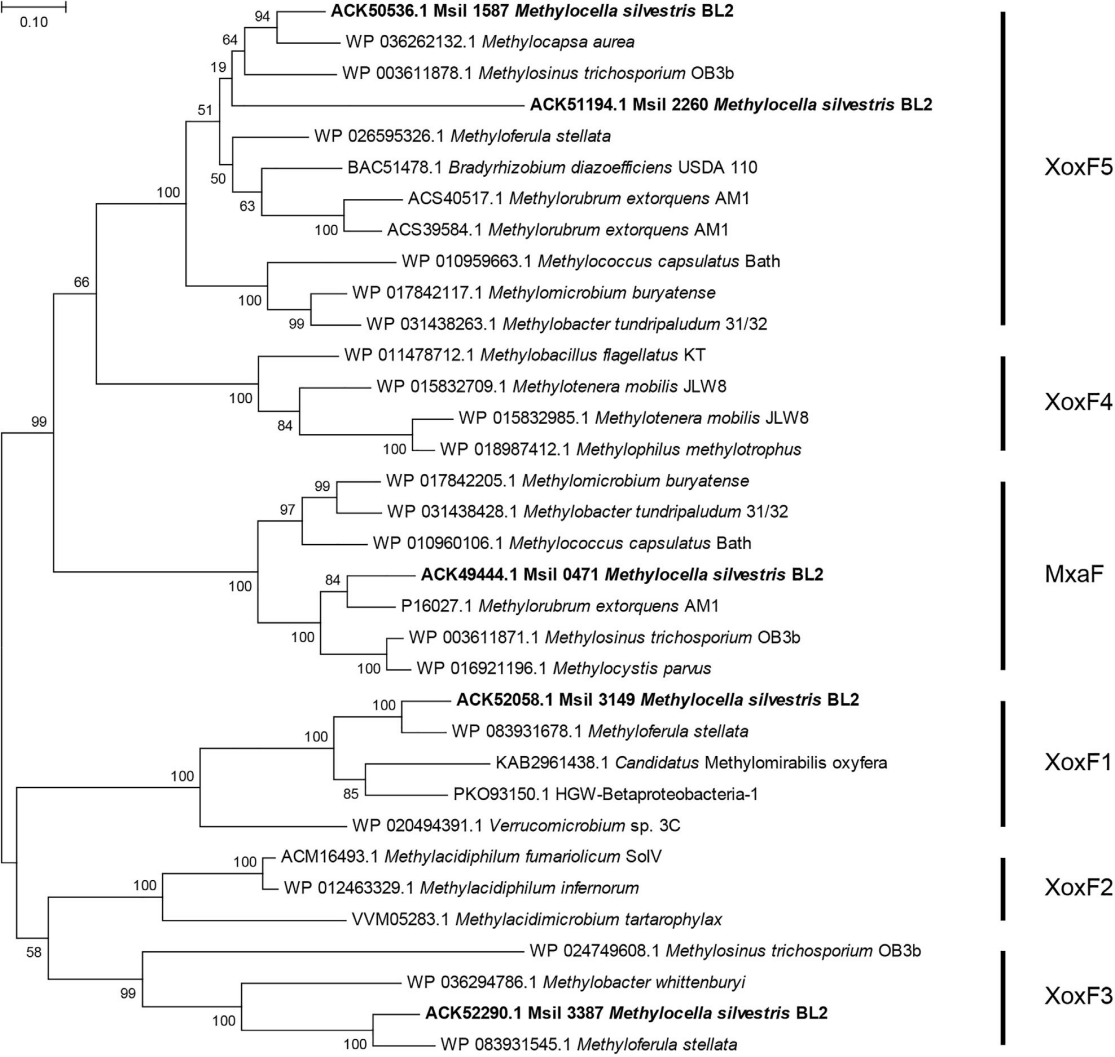
Relationship, based on amino acid sequences, of the putative *M. silvestris* MDHs (in bold type) with those of representative examples from the databases. The tree was constructed using the maximum likelihood method in MEGA 7 (Kumar *et al*., 2016). Gaps were eliminated, there were 566 positions in the final dataset and the scale bar shows substitutions per site. Bootstrap values (500 replications) are shown at the nodes.

**Figure 2:**
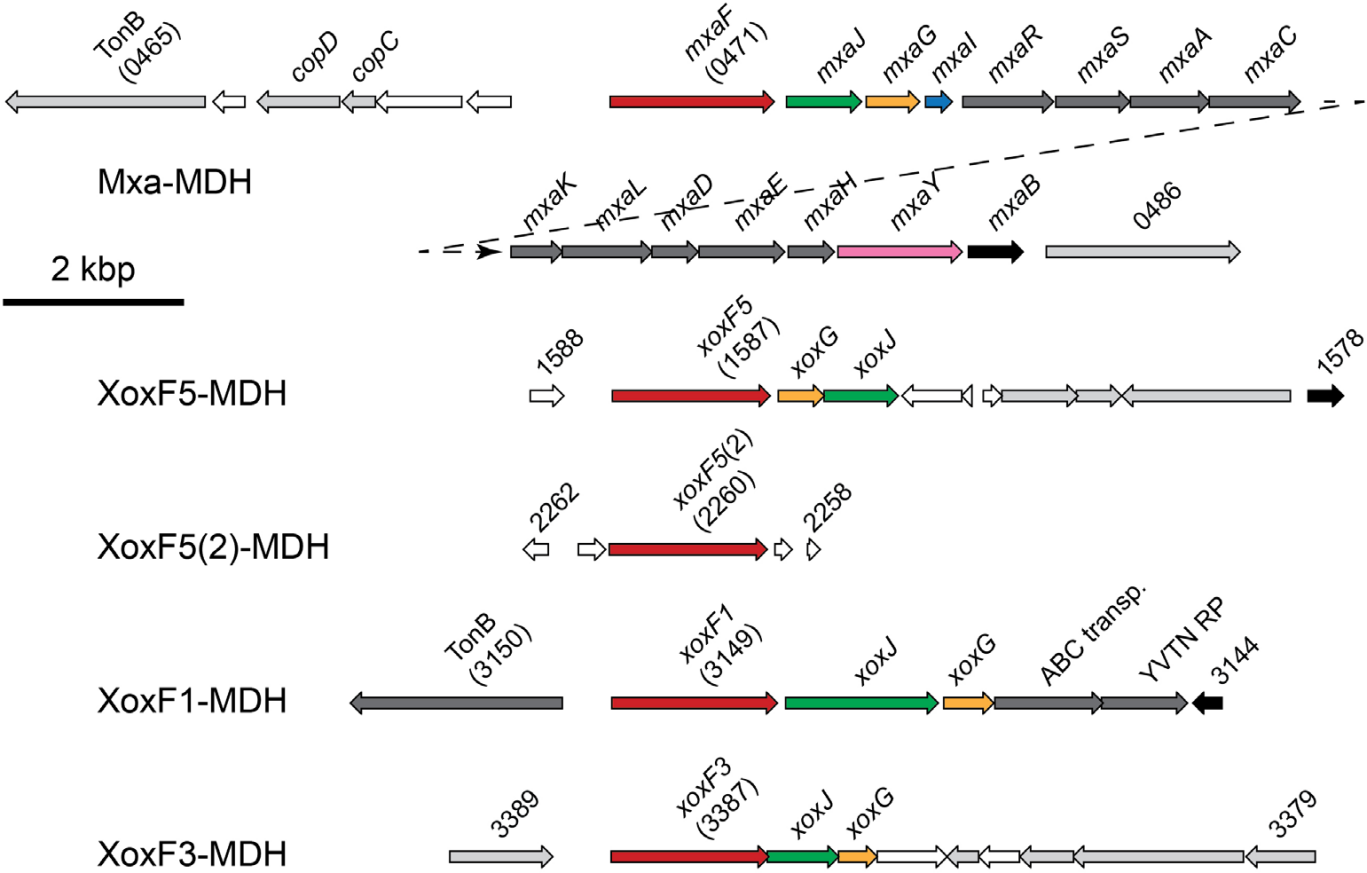
MDH gene clusters in *M. silvestris*. The genes are colour coded: red, MDH large subunit; blue, small subunit; yellow, cytochrome c; green, solute binding protein; pink, sensor histidine kinase; black, response regulators; dark grey, other MDH‐associated genes; light grey, no direct MDH association; white, hypothetical proteins of unknown function. Four‐digit gene identifications refer to abbreviated locus tags, e.g. 0471, Msil_0471 (*mxaF*). YVTN RP, 40 residue YVTN repeat protein; TonB, TonB‐dependent receptor.

**Figure 3:**
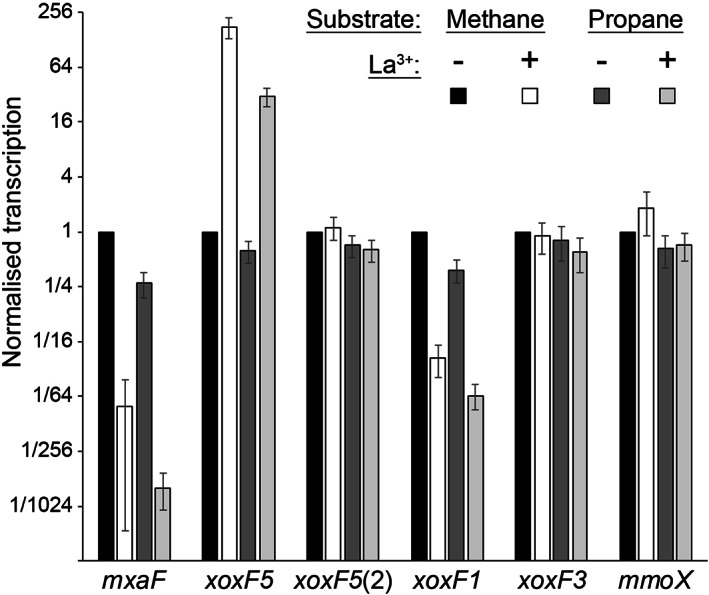
MDH gene transcription in cells grown on methane or propane in the presence or absence of Ln. Transcript levels (fold‐change, normalized to reference gene *rpoB*) are shown relative to growth on methane without Ln. Error bars show the standard error of the mean (*n* = 3)

The *M. silvestris* genome contains five predicted PQQ‐dependent MDH genes, Msil_0471, Msil_1587, Msil_2260, Msil_3149 and Msil_3387. Based on amino acid sequence and the presence of the Ln‐coordinating aspartate residue in Xox‐MDH, these can be identified as encoding Mxa‐ and Xox‐MDHs from clades 5 (two copies), clade 1 and clade 3 (Fig. 1 and Supporting Information Fig. S1), herein referred to as MxaF, XoxF5, XoxF5(2), XoxF1 and XoxF3, respectively. The genome also contains two predicted PQQ‐ADH sequences, Msil_2867 and Msil_3733, clustering with group 9 PQQ‐ADH (Keltjens *et al*., 2014), both of which also contain the Ln‐coordinating Asp residue, but whose function is unknown. Despite the fact that *M. silvestris* grows well on ethanol, no other sequences related to characterized PQQ‐dependent ethanol dehydrogenases were found, such as ExaF from *Methylorubrum extorquens* or PedE/H from *Pseudomonas putida* (Good *et al*., 2016; Wehrmann *et al*., 2017). Examination of the genome sequence in the vicinity of each of these putative MDH genes revealed the presence of the MDH‐associated cytochrome *c*_*L*_ (*xoxG*) and solute‐binding protein (*xoxJ*), except for the second *xoxF5* copy, which lacks any other recognizable MDH‐related sequences nearby (Fig. 2). Since these Ca^2+^‐ and Ln^3+^ ‐dependent MDHs were expected to be transcriptionally regulated by the presence of Ln, cells were grown in the absence or presence of Ln and gene expression levels were evaluated by RT‐qPCR (Fig. 3). During growth on methane, the addition of Ln (10 μM) to cultures increased transcription of *xoxF5* and repressed *mxaF* 180‐ and 80‐fold respectively. Transcription of *xoxF5*(2) and *xoxF3* was not significantly affected by the addition of Ln, and the transcription of these two genes appeared to be at a low level based on the cycle threshold (Ct) values. Interestingly, in contrast to *xoxF5*, *xoxF1* was downregulated 24‐fold by the addition of Ln, thus responding similarly to *mxaF*, despite the presence of the predicted Ln‐coordinating aspartate residue (Supporting Information Fig. S1). It is interesting to note that the XoxF (clade 1) purified from *Candidatus* Methylomirabilis oxyfera appeared to be a calcium‐containing enzyme (Wu *et al*., 2015). If this is also the case for the *Methylocella* protein (68% amino acid identity with the sequence from *Ca*. M. oxyfera), downregulation in response to Ln might be expected.

Since *M. silvestris* can grow on propane, which is oxidized to both 1‐propanol and 2‐propanol (Crombie and Murrell, 2014), MDH transcription was also assessed during growth on propane, and the same pattern of MDH regulation was observed (Fig. 3). Transcription of *mmoX*, encoding the sMMO alpha subunit and included as an additional control, was not significantly affected, either by growth substrate (as expected, Crombie and Murrell, 2014) or by the addition of Ln. Hence, the lanthanide switch operates in *Methylocella*, as has been previously observed in both non‐methane‐utilizing methylotrophs and obligate methanotrophs, see Skovran *et al*. (2019) for a review.

### Growth phenotype in the presence or absence of lanthanum

**Figure 4:**
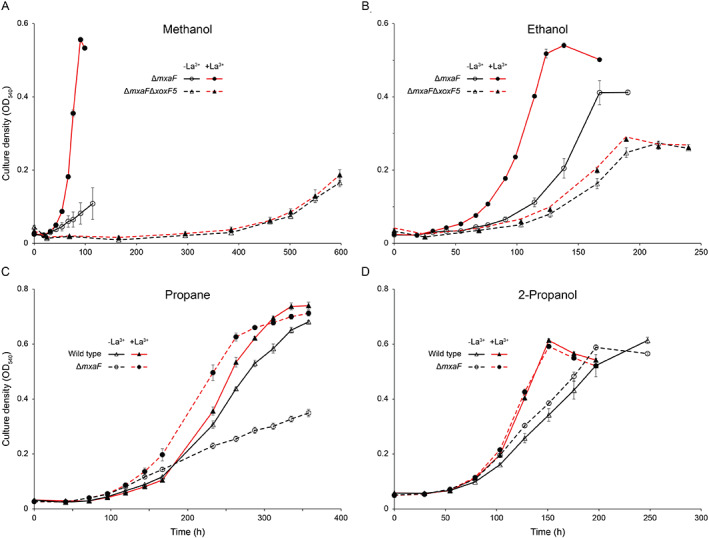
Upper panels, growth of strain Δ*mxaF* (solid lines) and of double mutant Δ*mxaF*Δ*xoxF5* (dashed lines) on (A) methanol (0.1% v/v) or B) ethanol in the absence (black) or presence (red) of Ln (5 μM). Lower panels, growth of the wild type (solid lines) and strain Δ*mxaF* (dashed lines) on (C) propane (20% v/v) or (D) 2‐propanol (0.05% v/v) in the absence (black) or presence (red) of Ln (5 μM). Error bars show the standard error of the mean (*n* = 3).

*M. silvestris* grew slightly better on methanol in the presence compared to the absence of Ln (5 μM) (approximately 6% improvement in growth rate, Table 1; Supporting Information Fig. S2A). Since this indicated an effect of added Ln, unmarked mutants were constructed with deletions of the MDH genes regulated by Ln: *mxaF*, *xoxF5*, and *xoxF1* and double mutants in *mxaF/xoxF5* and *mxaF/xoxF1*, and the growth phenotype of the mutants was assessed. The presence or absence of Ln made no difference to growth on pyruvate, and strain Δ*mxaF* grew similarly to the wild type on methanol in Ln‐containing medium, but at approximately one quarter of the rate in the absence of Ln, and without achieving exponential growth (Table 1, Fig. 4A), clearly showing the importance of the Ca‐containing enzyme in medium without Ln, but implying that another enzyme is able to substitute, to a certain extent, for Mxa‐MDH. One possible candidate might be XoxF1, transcriptionally regulated in the same sense as *mxaF*. However, Ln had a very similar effect on growth of mutant strain Δ*xoxF1* as it did in the wild type, i.e. a 3% increase in growth rate in the presence compared to absence of Ln, implying at most a minor role for XoxF1 during growth on methanol, also confirmed by the phenotype of double‐mutant Δ*mxaF*Δ*xoxF1*, which closely resembled that of strain Δ*mxaF* (Table 1). Alternatively, it might be that XoxF5 was sufficiently expressed and active in the absence of supplied Ln, for example, if cells were able to scavenge sufficient Ln from glassware (despite precautions), or if the enzyme had sufficient levels of expression and activity in the absence of Ln, to enable this comparatively low growth rate. However, although double‐mutant Δ*mxaF*Δ*xoxF5* did not grow discernibly for approximately 300 h, it subsequently grew at approximately 13% of the wild‐type rate (Fig. 4A), irrespective of the presence or absence of Ln. Due to the technical difficulties experienced in construction of these double mutants, construction of a triple *mxaF/xoxF1/xoxF5* mutant was not attempted. However, the lack of a distinct phenotype for strain Δ*xoxF1* during growth on methanol suggests that a triple mutant would behave similarly to strain Δ*mxaF*Δ*xoxF5* under the experimental conditions described here. Together, these data suggest that the combined activity of XoxF5 and XoxF1 was not solely responsible for residual methanol oxidation in strain Δ*mxaF* without Ln, or in strain Δ*mxaF*Δ*xoxF5*, and that other non Ln‐dependent methanol‐oxidizing enzyme(s) were expressed under these conditions (discussed below).

**Table 1:** Specific growth rate (h^−1^ ± standard error of the mean) of *M. silvestris* strains grown on methanol, ethanol, propane or 2‐propanol in the absence or presence of lanthanum.

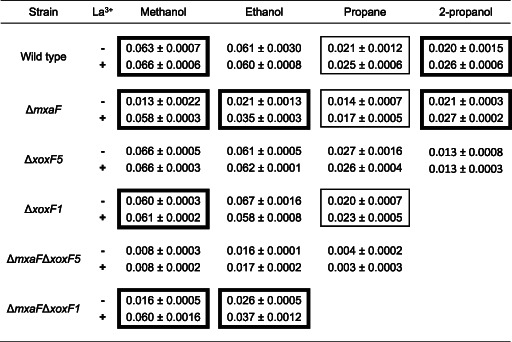

Significant differences of each strain/substrate combination in the absence of presence of lanthanum are shown with wide borders (*P* ≤ 0.01) or narrow borders (*P* ≤ 0.05). All cultures in triplicate except: wild type, methanol, *n* = 9; strain Δ*xoxF5*, methanol, *n* = 6; strain Δ*xoxF5*, ethanol, +La^3+^, *n* = 2. For strain Δ*mxaF*Δ*xoxF5*, the growth rates shown with methanol and propane are following extended lag phases of 300 and 600 h, respectively, as described in the text.

Finally, however, and perhaps surprisingly given the transcriptional switch from *mxaF* to *xoxF5* induced by Ln, strain Δ*xoxF5* grew at wild‐type rates irrespective of the presence or absence of Ln (Table 1).

### Promoter activity

**Figure 5:**
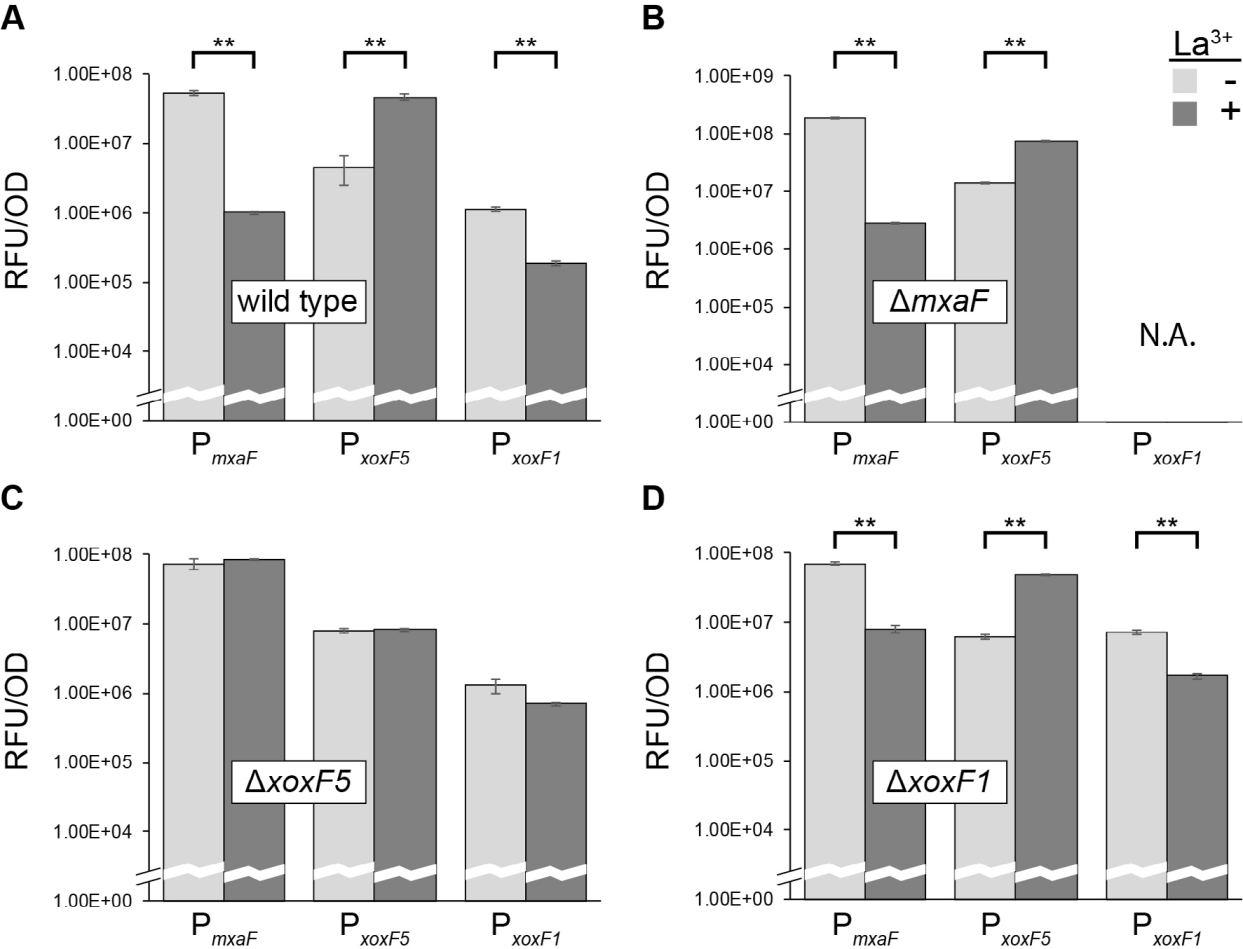
MDH promoter activity of wild‐type and mutant strains during growth on methanol. The bar chart shows background‐subtracted relative fluorescent units (RFU), normalized to cell density, of (A), wild‐type strain; (B), strain Δ*mxaF*; (C), strain Δ*xoxF5* and (D), strain Δ*xoxF1*, containing reporter constructs consisting of a green fluorescent protein (*gfp*) gene under the control of the *mxaF*, *xoxF5*, or *xoxF1* promoters, grown in the presence or absence of Ln (5 μM). Error bars show the standard error of the mean (*n* = 3) and significant differences between strains grown with or without Ln are shown as ** (*P* ≤ 0.01). N.A., not assayed.

This lack of a Ln‐dependent growth phenotype in strain Δ*xoxF5* suggested either, that under these conditions an alternative enzyme was responsible for methanol oxidation, or that deletion of *xoxF5* interfered with the differential transcriptional regulation of *mxaF* and *xoxF5* described above (as has been observed previously in other studies; Chu and Lidstrom, 2016; Zheng *et al*., 2018; Groom *et al*., 2019; Ochsner *et al*., 2019; Yanpirat *et al*., 2020). Therefore, following the strategy of these earlier researchers, the activity of the promoters of the MDH genes was assessed using promoter‐reporter plasmids, consisting of DNA fragments upstream of *mxaF*, *xoxF5* and *xoxF1* (952, 619 and 588 bp respectively) cloned in front of a *gfp* gene (encoding green fluorescent protein, GFP), which were introduced into the wild type and the single deletion strains. These GFP‐expressing reporter strains were then grown on methanol in the presence or absence of Ln and fluorescence was quantified (Fig. 5). In the wild type, promoter activity mirrored the RT‐qPCR data shown in Fig. 3, with the *mxaF* and *xoxF1* promoter activities reduced 53‐ and 6‐fold, respectively, in the presence compared to the absence of Ln, whereas *xoxF5* promoter activity was increased 10‐fold. This same Ln‐dependent promoter activity was evident in mutant strains Δ*mxaF* and Δ*xoxF1*. In contrast, in strain Δ*xoxF5*, the promoter activities of *mxaF* and *xoxF5* were entirely unaffected by Ln while only a minor (not statistically significant) difference in the activity of the *xoxF1* promoter was detected. While confirming the operation of the lanthanide switch in the wild type shown by the RT‐qPCR results above, these data show that this transcriptional control of *mxaF* and *xoxF5* (and likely *xoxF1*) was disabled by deletion of *xoxF5*, suggesting either that the XoxF*5* protein is itself involved in gene regulation, as shown previously (Skovran *et al*., 2011; Good *et al*., 2020), and/or that a suppressor mutation developed following gene deletion, allowing transcription of *mxaF* in the presence of Ln. It was previously shown in both alphaproteobacterial methylotrophs and gammaproteobacterial methanotrophs that suppressor mutations occurred in sensor kinases likely involved directly or indirectly in Ln sensing, thus allowing transcription and expression of Mxa‐MDH in the presence of Ln (Chu and Lidstrom, 2016; Zheng *et al*., 2018; Ochsner *et al*., 2019; Yanpirat *et al*., 2020). Genes encoding a two‐component system comprising sensor histidine kinase MxaY (Msil_0484) and response regulator MxaB (Msil_0485) are located at the end of the Mxa‐MDH gene cluster in *M. silvestris*. This arrangement is present in several alphaproteobacterial methanotrophs including *Methylosinus* and *Methylocystis* strains but differs from *Methylorubrum extorquens* AM1, which contains only an orphan response regulator *mxaB* at this locus (Springer *et al*., 1998). Despite the low sequence identity between *M. silvestris* MxaY and homologues in the databases (max 43% amino acid identity with examples outside the family Beijerinckiaceae), the location of the *mxaY* gene, adjacent to Mxa‐MDH, suggested that a mutation might have occurred in this gene. Sequencing of the PCR products, using primers targeting the entire *mxaY* gene in the wild type and strain Δ*xoxF5*, revealed that compared with the wild type, all mutant sequences (6/6) had a deletion after nucleotide 402, resulting in a predicted truncated protein of 172 amino acids (compared with 473 in the wild type). While these data do not prove a connection between *mxaY* mutation and *mxaF* expression in the presence of Ln, they suggest a role for MxaY in the lanthanide switch in *M. silvestris*. Interestingly, strain Δ*xoxF5* never exhibited a Ln‐dependent phenotype, did not appear to adapt over time to growth in the presence of Ln and grew in Ln‐containing medium without an extended lag phase, suggesting that the suppressor mutation was generated immediately and illustrating the strong selective pressure for efficient methanol oxidation in this strain.

### Growth on ethanol

The effect of Ln on *M. silvestris* while growing on ethanol resembled its effect during growth on methanol, except that the addition of Ln made no difference to the growth rate of the wild type (Table 1; Supporting Information Fig. S2B). As before, strain Δ*mxaF* exhibited a severe growth defect without Ln, which was partially alleviated by addition of Ln. Double‐mutant Δ*mxaF*Δ*xoxF5* was capable of relatively poor growth irrespective of the presence or absence of Ln, albeit at a low growth rate and to a comparatively low final cell density, but without the extremely long lag phase noticeable during growth on methanol (Fig. 4B). Furthermore, unlike on methanol, strain Δ*mxaF* achieved exponential rather than linear growth on ethanol in the absence of Ln. As was the case for methanol, the presence or absence of Ln had no effect on the growth of strain Δ*xoxF5* on ethanol, which grew at similar rates to the wild type under both conditions, and double‐mutant Δ*mxaF*Δ*xoxF1* behaved similarly to strain Δ*mxaF* (Table 1). These data demonstrate that the lanthanide switch also operates during growth on ethanol and confirm that not only methanol‐oxidizing activity but also ethanol‐oxidizing activity exists in cells lacking both principal MDHs, irrespective of the presence of Ln.

### Growth on propane and 2‐propanol

Since *M. silvestris* can also grow on propane, which is oxidized by *M. silvestris* to both 1‐propanol and 2‐propanol (although the strain does not grow on 1‐propanol; Crombie and Murrell, 2014), and MDH transcription was regulated by Ln during growth on propane, the response of the wild type and mutant strains to Ln was tested during growth on propane. Notably, the wild type, strain Δ*mxaF* and strain Δ*xoxF1* all grew significantly better (16%–22% increase in growth rate) on propane in the presence compared to the absence of Ln (Table 1, Fig. 4C), demonstrating a Ln‐dependent phenotype. As before, deletion of XoxF5 removed the effect of Ln on growth on propane, confirming that a similar mode of regulation of these enzymes operates during growth on propane as during growth on 1‐ and 2‐carbon substrates. Double‐mutant Δ*mxaF*Δ*xoxF5* eventually grew at approximately 16% of the wild‐type rate, but only after an extremely long lag phase of approximately 600 h, during which there was no growth whatsoever, again suggesting the eventual expression of an alternative enzyme in the absence of MDH. Growth on 2‐propanol, one of the products of propane oxidation, was also evaluated with and without Ln. The wild type and strain Δ*mxaF* behaved in an almost identical manner and addition of Ln stimulated growth almost identically in both strains (27%– 34% increase in growth rate, Fig. 4D), whereas strain Δ*xoxF5*, which was not affected by Ln, grew at half the rate of the Ln‐supplied wild type (Table 1). These data suggest a minor role for Mxa‐MDH in 2‐propanol oxidation and that Xox‐MDH was more efficient in oxidation of this secondary alcohol. There was a clear Ln‐mediated growth advantage to cells growing on both propane and 2‐propanol and the results suggest that the benefit of Ln during growth on propane is at least partly due to enhanced oxidation of 2‐propanol. While Ln‐dependent enzymes capable of oxidation of secondary alcohols have been described, in general Ln‐dependent enzymes for which methanol is not the primary substrate are distinct from Xox‐MDH and have comparatively poor catalytic properties with methanol (Keltjens *et al*., 2014; Good *et al*., 2016; Wehrmann *et al*., 2017; Huang *et al*., 2018; Picone and Op den Camp, 2018; Wang *et al*., 2019). Hence, the finding that *M. silvestris* uses the same pair of versatile enzymes, one dependent on Ca and one on Ln, for oxidation of C_1_–C_3_ primary and secondary alcohols is both novel and surprising.

### Protein expression

**Figure 6:**
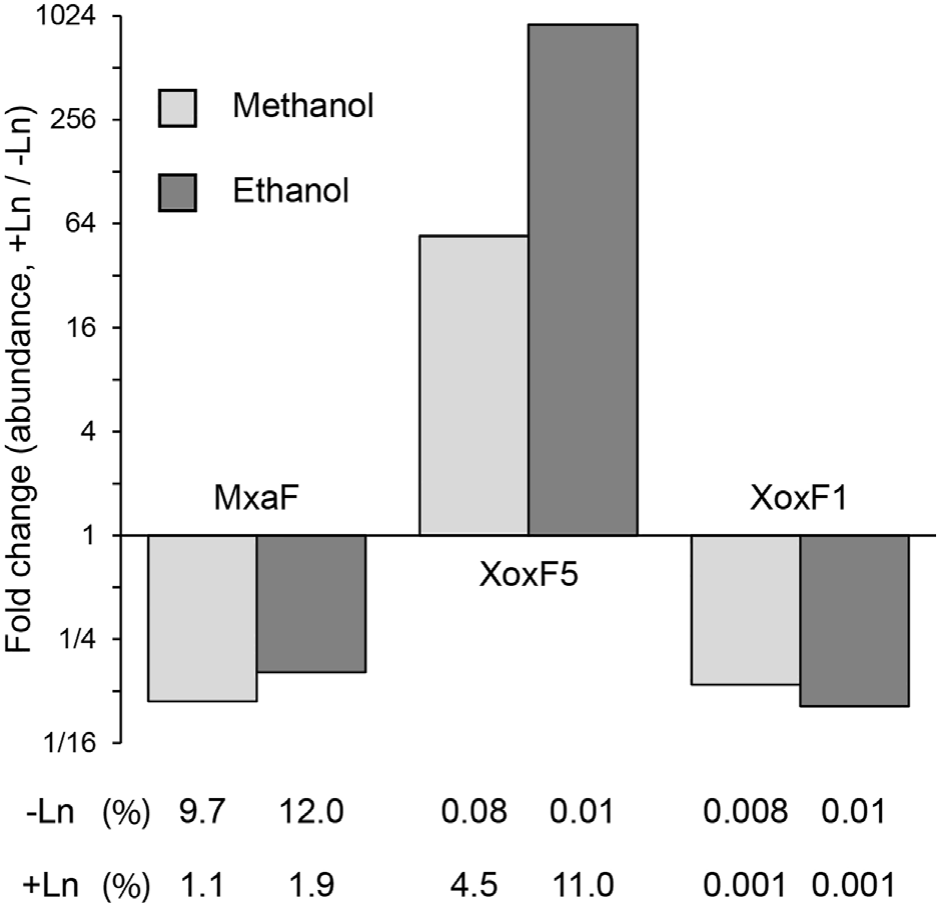
Change in abundance of MDH proteins in wild type *M. silvestris* cells grown on methanol or ethanol in the presence Ln, compared with growth without Ln. The figures below the bars show the abundance of each protein in the presence or absence of Ln as percentage of total protein.

To observe how gene transcription was correlated with protein expression and to examine the wider impact of the lanthanide switch, protein was extracted from cells grown on methanol or ethanol with or without Ln and the proteomes were determined by liquid chromatography/mass spectrometry (LC/MS). Since double‐mutant strains Δ*mxaF*Δ*xoxF5* and Δ*mxaF*Δ*xoxF1* were able to grow to a certain extent on all substrates tested, both with and without Ln, albeit, in some cases, with a long lag phase, these strains were included in the proteomic analysis. Strain Δ*mxaF*Δ*xoxF5* was grown on methanol or ethanol with or without Ln, and strain Δ*mxaF*Δ*xoxF1* was grown on methanol or ethanol, without Ln only, resulting in 10 strain/condition combinations. A total of 2472 proteins were identified by two or more unique peptides. As predicted from transcriptional data, of the five predicted PQQ‐dependent MDHs, only MxaF, XoxF5 and XoxF1 were expressed at appreciable levels. XoxF5(2) (Msil_2260) was not detected in any sample, and XoxF3 (Msil_3387) was expressed at very low levels ranging from 0.00003% to 0.00017% of total protein, Supporting Information Table S1. In comparison, MxaF and XoxF5 were among the most abundant proteins in the wild type, comprising 9.7% and 4.5% of the total during growth on methanol in the absence or presence of Ln respectively (Fig. 6). XoxF1 was expressed at comparatively low levels, three orders of magnitude less than MxaF. To investigate the effect of Ln on protein expression, proteins expressed by cells grown on methanol in the presence of Ln were compared with proteins expressed in its absence, and the same comparison was made with cells grown on ethanol. In total, 44 proteins were differentially expressed (at least fourfold, *P* ≤ 0.01) during wild‐type growth on methanol with/without Ln, of which 9 were upregulated and 35 downregulated in the presence of Ln (Supporting Information Table S1). During growth on ethanol, 49 proteins were similarly differentially expressed, of which 23 were upregulated. The Ln‐induced regulation of MDH proteins, similar for growth on both methanol and ethanol (Table 2), reflected the RT‐qPCR transcriptional data. During growth on methanol, in comparison with the Ln‐free condition, the presence of Ln induced 54‐fold higher expression of XoxF5 (Fig. 6) and the XoxF5‐associated cytochrome *c*_*L*_ and SBP (XoxG and XoxJ) were also up‐regulated (ninefold and sevenfold, Table 2). In contrast, MxaF was downregulated ninefold in Ln‐containing medium, together with the associated proteins of the Mxa‐MDH operon (Msil_0472–0485), downregulated between sixfold and 28‐fold. Interestingly, and as predicted by RT‐qPCR data, XoxF1 (Msil_3149) was also downregulated (sevenfold) by Ln, together with its associated cytochrome *c*_*L*,_ XoxG (Msil_3147) and solute‐binding protein XoxJ (Msil_3148) (sevenfold and 62‐fold, respectively). Of the Xox‐MDH enzymes, clades 5, 4 and 2 have been most well characterized (Picone and Op den Camp, 2018), with the exception of the XoxF clade 1 enzyme purified from the anaerobe *Ca*. Methylomirabilis oxyfera (Wu *et al*., 2015). This unusual methanotroph contains a single cluster of genes predicted to encode three MDH homologues, Mxa‐MDH and two Xox enzymes from clades 1 and 2. The XoxF1‐MDH from *Ca*. M. oxyfera was purified with high activity and surprisingly seemed to contain Ca rather than Ln (as mentioned above), despite the presence of the diagnostic Ln‐coordinating aspartate residue (D^305^ in the XoxF5 from *Methylorubrum extorquens*), also present in *M. silvestris* XoxF1 (Supporting Information Fig. S1) (Keltjens *et al*., 2014; Wu *et al*., 2015; Good *et al*., 2020). In *M. silvestris*, the mutant phenotypes suggested that XoxF1 plays a minor role under the experimental conditions used here, but XoxF1 was clearly down‐regulated, at both RNA and protein level, by Ln in the wild type, perhaps suggesting that this enzyme might also contain Ca rather than Ln. Therefore, more work is needed to determine the role and properties of this enzyme in *M. silvestris*.

**Table 2:** Relative abundance of MDH proteins and proteins thought to be involved in the lanthanide switch in wild‐type *M. silvestris* grown on methanol or ethanol in the presence compared with the absence of lanthanum.

		Methanol		Ethanol	
		log_2_ (FC)	*P* value	log_2_ (FC)	*P* value
**Mxa‐MDH**					
Msil_0471	MxaF (large s/u)	−3.2	2.8E‐12	−2.6	1.2E‐03
Msil_0472	MxaJ (SBP)	−4.8	2.3E‐15	−4.2	8.6E‐10
Msil_0473	MxaG (Cyt c)	−3.9	2.3E‐15	−2.9	2.6E‐04
Msil_0475	MxaR	−3.1	5.2E‐12	−2.5	2.2E‐03
Msil_0476	MxaS	−3.4	4.8E‐14	−2.4	4.1E‐03
Msil_0482	MxaE	−2.4	7.6E‐07	−2.9	2.8E‐04
Msil_0483	MxaH	−3.2	4.3E‐12	−2.7	7.6E‐04
Msil_0485	MxaB (RR)	−4.1	2.3E‐15	−3.4	6.2E‐06
**XoxF5‐MDH**					
Msil_1585	XoxJ5 (SBP)	2.5	5.6E‐07	3.4	1.5E‐06
Msil_1586	XoxG5 (Cyt c)	3.1	5.0E‐11	4.2	3.1E‐10
Msil_1587	XoxF5 (large s/u)	5.8	2.3E‐15	9.8	7.0E‐15
**XoxF1‐MDH**					
Msil_3147	XoxG1 (Cyt c)	−2.7	4.3E‐09	−2.5	3.0E‐03
Msil_3148	XoxJ1 (SBP)	−6.0	2.3E‐15	−4.5	4.8E‐11
Msil_3149	XoxF1 (large s/u)	−2.9	5.1E‐10	−3.3	1.1E‐05
**Others**					
Msil_0271	LanM	5.3	2.3E‐15	4.7	4.1E‐13
Msil_3146	ABC‐transporter	−2.2	4.0E‐06	−2.8	5.0E‐04

The data show the log_2_ (fold‐change, FC) of proteins upregulated or downregulated ≥ 4‐fold (|log_2_| ≥ 2 and *P* ≤ 0.01), in both the methanol and ethanol growth condition, together with adjusted *P* values. Cells are shaded to show upregulation and downregulation (red and blue, respectively).

As well as the MDHs, also highly regulated was lanmodulin (LanM, Msil_0271), 39‐ and 26‐fold upregulated by Ln in methanol‐ and ethanol‐grown cells respectively (Table 2). Lanmodulins, which bind lanthanides, are recently discovered EF‐hand domain‐containing homologues of the well‐studied calcium‐binding calmodulin (Featherston and Cotruvo, 2021). LanM was proposed to be a periplasmic receptor for Ln transported through the outer membrane by a TonB transporter (Cotruvo *et al*., 2018). Upregulation of LanM in the presence of Ln is consistent with its proposed role as a periplasmic shuttle of Ln to Xox‐MDH (Cotruvo *et al*., 2018) and these data support similar findings of Ochsner *et al*. (2019), although, interestingly, the *lanM* gene was not differentially expressed when the transcriptome of *Methylorubrum extorquens* AM1 was examined in response to Ln (Good *et al*., 2019), and close homologues of this gene do not appear to be present in several gammaproteobacterial methanotrophs. In *Methylorubrum*, this gene is located in a 10‐gene cluster encoding proteins including a TonB receptor, components of an ABC transport system and other periplasmic proteins of unknown function, predicted to be responsible for Ln transport (Ochsner *et al*., 2019; Roszczenko‐Jasińska *et al*., 2020). Although *M. silvestris* has homologues of several of these genes, they are not co‐located with *lanM*. Three of them, encoding a YVTN family beta‐propeller repeat protein, an ABC‐transporter substrate‐binding protein and a TonB receptor (homologues of LutBAH as defined in the study by Roszczenko‐Jasińska *et al*., 2020) are adjacent to the *xoxF1* genes (Fig. 2) and were moderately downregulated (threefold to fivefold) by Ln (Supporting Information Table S1). The TonB receptor shares 40% amino acid identity with a *Methylosinus trichosporium* OB3b gene product (WP_003609830.1), which was similarly transcriptionally downregulated in response to cerium (Gu and Semrau, 2017). Hence, in *M. silvestris*, under these conditions, expression of these proteins was regulated differently than expression of LanM. The Ln concentration (5 μM) used here is orders of magnitude higher than the bio‐available concentrations commonly encountered in the environment (Glass *et al*., 2020) suggesting that toxicity (due to mis‐metalation) might be important. Under these circumstances, it might be an appropriate strategy to reduce membrane transport and increase abundance of LanM to minimize availability of free Ln^3+^.

Apart from MDH, MDH‐associated proteins and Ln trafficking proteins, only two other proteins were differentially expressed during growth on both methanol and ethanol: the RNA chaperone Hfq and a subunit of nitrate reductase (Supporting Information Table S1), although several other enzymes associated with nitrogen metabolism were regulated during growth on methanol (but not ethanol). Hfq is an RNA‐binding protein with a role in global regulation of gene expression (Vogel and Luisi, 2011). Although a direct role for Ln in nitrogen metabolism cannot be ruled out, and expression of nitrogen metabolism genes in *Methylosinus trichosporium* varied depending on metal availability (Gu and Semrau, 2017), it seems more likely that these changes are indirect results of the lanthanide switch, possibly due to Ln‐mediated changes in growth rate or carbon utilization efficiency, or perhaps due to differences in the product distribution (formaldehyde or formate) of the Ca‐ and Ln‐containing MDH enzymes and consequent metabolic re‐programming (Keltjens *et al*., 2014).

As described above, the expression pattern of MDHs was similar during growth on both methanol and ethanol (Fig. 6; Supporting Information Tables S1 and S2). *Methylorubrum extorquens* expresses a Ln‐dependent ethanol dehydrogenase, ExaF, that contributes to ethanol oxidation and is also able to sustain growth on methanol in MDH knockouts (Good *et al*., 2016). On the other hand, comparison of gene transcription in the facultative methanotroph *Methylocystis* sp. SB2, grown on methane or ethanol, showed that the switch to ethanol repressed *mxaF* transcription 49‐fold and induced transcription of an NAD(P)‐dependent short‐chain dehydrogenase (Vorobev *et al*., 2014), suggesting that Mxa‐MDH may not be the primary enzyme responsible for ethanol oxidation in this *Methylocystis* strain. However, in *M. silvestris*, no other predicted alcohol dehydrogenases were highly expressed and the abundance of MxaF or XoxF5 increased when cells were grown on ethanol in comparison to on methanol (Fig. 6). Together with growth data presented above, these data show that *M. silvestris* uses the same Mxa/Xox enzymes to oxidize both methanol and ethanol and that the lanthanide switch operates with both substrates.

**Figure 7:**
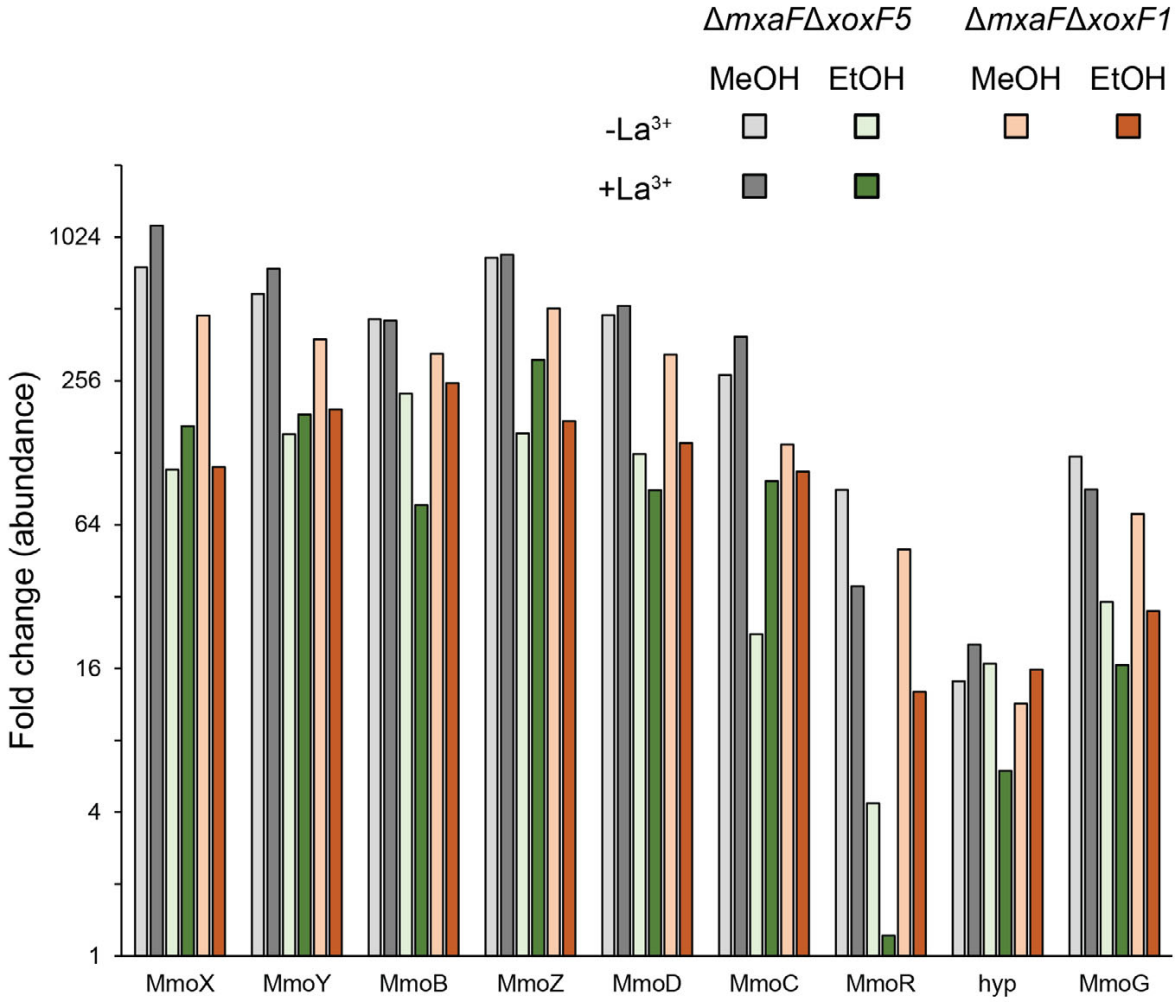
Relative abundance of the sMMO in mutant strains compared with the wild type. The sMMO structural polypeptides (MmoXYBZDC) were upregulated 22‐ to over 1000‐fold, with more modest levels for the regulatory protein and chaperone (MmoRG) in mutant strains Δ*mxaF*Δ*xoxF5* and Δ*mxaF*Δ*xoxF1* during growth on methanol or ethanol, irrespective of the presence of Ln, compared with the wild type. All *P*‐values ≤ 0.01, see Supporting Information Table S4 for full details. hyp, hypothetical protein of unknown function.

To investigate which alcohol‐oxidizing enzyme(s) enabled the comparatively slow growth of double‐mutants Δ*mxaF*Δ*xoxF5* and Δ*mxaF*Δ*xoxF1*, proteins upregulated in these strains in comparison with the wild type under the same growth conditions were identified. Several proteins (19–73) were regulated (≥4‐fold, *P* ≤ 0.01) in mutant Δ*mxaF*Δ*xoxF5* during growth on both methanol and ethanol, either without or with Ln, and in mutant Δ*mxaF*Δ*xoxF1* during growth on these substrates without Ln, in comparison with the wild type under the same conditions, indicative of disrupted core metabolic processes (Supporting Information Table S4). None of these are annotated with a possible short‐chain alcohol‐metabolizing ability or are homologues of short‐chain alcohol dehydrogenases, suggesting that no alternative alcohol dehydrogenases took the place of PQQ‐MDH. In particular, XoxF1 was not upregulated in strain Δ*mxaF*Δ*xoxF5*. Instead, all the mutant strain/growth conditions expressed the soluble methane monooxygenase (sMMO) polypeptides. The wild type does not express the sMMO at high levels during growth on methanol or ethanol, but in these mutants, the structural polypeptides were upregulated 22‐ to over 1000‐fold over the corresponding wild‐type expression levels (Fig. 7), approximating the levels of the wild type during growth on methane (Patel *et al*., 2012), with the sMMO comprising 14%–27% of total protein in mutants cells. The sMMO has a well‐documented wide substrate range, including methanol and ethanol (Colby *et al*., 1977; Patel *et al*., 1982; Tinberg and Lippard, 2010), suggesting that suppressor mutations may have arisen in the sMMO regulatory network (of which little is known in *Methylocella*), to allow sMMO‐mediated alcohol oxidation. To verify that the sMMO was responsible for the eventual growth of double‐mutant strain Δ*mxaF*Δ*xoxF5* on methanol, cells were subcultured into methanol‐containing medium during active growth and incubated in the presence or absence of the sMMO inhibitor, acetylene. Cells incubated with acetylene did not grow during the 640 h of the experiment, whereas without acetylene cells grew at the low growth rate previously observed with this strain. In contrast, acetylene had little or no effect on growth of the wild type on methanol (Supporting Information Fig. S2) demonstrating that the sMMO could enable weak growth on methanol in the absence of both MxaF and XoxF5.

## Conclusions

The participation of lanthanides in biological processes was only discovered recently, and there is still much to learn. The data presented here expand the breadth of our knowledge of Xox‐MDHs in the family Beijerinckiaceae, not previously studied in the context of Ln regulation, and show that the lanthanide switch operates in the facultative methanotroph *M. silvestris*. Lanthanides were first implicated in methanol metabolism and subsequently in ethanol metabolism (Hibi *et al*., 2011; Good *et al*., 2016) and the data presented here extend the role of Ln‐containing enzymes to growth on propane. *Methylocella* are environmentally important bacteria, which are extremely abundant in some environments where methane and other short‐chain alkanes co‐exist (Farhan Ul Haque *et al*., 2018; Farhan Ul Haque *et al*., 2019), suggesting that the ability to benefit from these multiple carbon sources may be important for its competitive success (Crombie and Murrell, 2014). In contrast to other ethanol‐oxidizing methylotrophs such as *Methylorubrum extorquens* and *Methylocystis* sp. SB2, however, *M. silvestris* expresses only the same pair of enzymes, depending on Ln availability, to oxidize not only C_1_ and C_2_ but also C_3_ alcohols, despite the fact that most Ca‐containing MDHs do not efficiently oxidize secondary alcohols such as 2‐propanol (Goodwin and Anthony, 1998). The data suggest that in *M. silvestris*, Xox‐MDH is more efficient than Mxa‐MDH at oxidizing 2‐propanol, explaining the increased benefit of Ln during growth on propane and 2‐propanol (21% and 34% increase in growth rate due to Ln, respectively) compared to during growth on methanol (6% increase) (Table 1).

Most work has focussed so far on only a few examples of Xox‐MDH, mainly from clades 4 and 5 (Huang *et al*., 2019). The finding here that the XoxF1 of *M. silvestris* is also regulated by Ln, but in the same sense as Mxa‐MDH rather than as XoxF5‐MDH, is significant. Enzymes from this clade are distributed among a relatively small number of diverse taxa, including alpha‐ and gammaproteobacteria, verrucomicrobia and members of the candidate phylum NC10 (Keltjens *et al*., 2014). However, under the experimental conditions used here, there was no prominent phenotype of strain Δ*xoxF1*, and therefore the role of this enzyme is not clear. The location of *xoxF1* in *M. silvestris* adjacent to putative Ln‐trafficking genes suggests a possible regulatory role, as has already been shown for XoxF clade 5 enzymes in *Methylorubrum* (Skovran *et al*., 2011; Vu *et al*., 2016; Ochsner *et al*., 2019), and this warrants further investigation.

Deletion of *xoxF5* resulted in a suppressor mutation in a sensor kinase‐encoding gene associated with the Mxa‐MDH gene cluster, *mxaY*, in line with several previous studies which have identified mutations in homologous genes in other organisms (Chu and Lidstrom, 2016; Zheng *et al*., 2018; Ochsner *et al*., 2019; Yanpirat *et al*., 2020). So far, this is the only gene predicted to be involved in MDH regulation in *M. silvestris*, but the limited data available suggest that regulation is enabled by the two component regulator comprising MxaY and MxaB, with plasma‐membrane‐located sensor kinase MxaY responsive to Ln (directly or more likely indirectly), in the periplasm, and the cognate response regulator MxaB controlling transcription of *mxaF* and *xox* genes, with the effect of repressing *mxa* and *xoxF1*, and activating *xoxF5* in the presence of Ln. This is broadly in line with the model proposed for the gammaproteobacterial methanotroph *Methylotuvimicrobium buryatense* 5GB1C (Groom *et al*., 2019), although we should note that there are several differences between Ln‐trafficking and the lanthanide switch in *M. buryatense* and in alphaproteobacteria.

Generation of a strain constitutively expressing the sMMO in double Mxa‐Xox‐MDH mutants was surprising and fortuitous and suggests that repression of sMMO expression by alternative carbon sources (such as methanol), while presumably distinct from the lanthanide switch, was disabled in these strains. Little is known about sMMO regulation in *Methylocella*, and analysis of the genetic basis of constitutive sMMO expression may provide insights into MMO regulation, the subject of further study in this laboratory.

In conclusion, this work has expanded our knowledge of Ln‐dependent microbiology and established base data for *Methylocella*, which now enable more detailed examination of the properties and regulation of Mxa‐ and Xox‐MDHs in this uniquely versatile and environmentally important organism.

## Methods

### Growth of Methylocella

*M. silvestris* BL2 and mutant strains were grown in dilute mineral salts (DNMS) medium in 120 ml glass vials as previously described (Crombie and Murrell, 2014). All media were made using ultrapure water (18 MΩ cm) produced using an Elga Purelab Classic or Elga Purelab Chorus 1 Life Scence water purification system (Veolia Water Technologies, High Wycombe, UK). For evaluation of growth, gene transcription and protein expression, special precautions were taken to minimize contamination with lanthanides. At the completion of each experiment, cultures were killed by autoclaving. The vials were extensively rinsed in water, filled with de‐ionized water and re‐autoclaved and then acid washed by soaking overnight in 10% v/v nitric acid, followed by repeated rinsing in ultrapure water. For growth in the presence of Ln, lanthanum chloride was added to 5 μM just before autoclaving. Vials were segregated into those that had been used with Ln and those without Ln, for the duration of the study. Gases were added to the headspace by injection through the septum and other substrates were added from sterile stock solutions to the concentrations indicated. For acetylene inhibition experiments, acetylene (4 ml) was injected through the septum to give a headspace concentration of approximately 4% v/v.

### RT‐qPCR analysis of gene transcription

Cells were grown in 25 ml volume on methane or propane (20% v/v), with or without lanthanum (10 μM), in triplicate and harvested at mid‐exponential phase (OD_540_ = 0.21–0.36). Aliquots of the culture (4 ml) were immediately treated with RNA Protect Bacteria reagent (Qiagen, Hilden, Germany) following the manufacturer's instructions and the pellet was stored at −80°C, prior to RNA extraction using a hot acid phenol method as previously described (Gilbert *et al*., 2000). Following two treatments with RNase‐free DNase and purification using an RNeasy kit (Qiagen), RNA was eluted in 40 μl water and quantified using a Qubit fluorometer (Invitrogen, Carlsbad, USA). Synthesis of cDNA used Superscript III reverse transcriptase (RT) (Invitrogen) with 100 ng RNA and random hexamers in 20 μl reactions, following the manufacturer's instructions, and including controls in which RT was omitted. Standard‐curve‐based quantitative PCR was conducted in 25 μl reactions in 96‐well plates with SensiFAST SYBR Hi‐ROX master mix (Bioline, London, UK), 400–800 nM of each primer and 2 μl of 1/10 dilution of cDNA template, using an Applied Biosystems StepOnePlus instrument (Applied Biosystems, Waltham, USA). Standards for each target gene and no‐RT controls were included on each plate and transcription was quantified with respect to *rpoB* as reference gene. Primer sequences are given in Supporting Information Table S3. Two technical replicates were analysed for each of three biological replicates for both substrates, with and without Ln. Amplification efficiency was between 85% and 105% for all targets, except *mmoX* (75%). Target gene transcription, normalized to *rpoB*, is shown relative to levels in cultures grown on methane, without Ln.

### Construction of mutant strains

Unmarked deletions of *mxaF*, *xoxF5* and *xoxF1* were generated as described previously (Crombie and Murrell, 2011). Since MDH genes were the deletion targets, *M. silvestris* cells were grown on pyruvate (8 mM) or succinate (5 mM). Briefly, removal of the gene of interest was accomplished by homologous recombination between arms A and B (DNA sequences identical to approximately 500 bp either side of the gene of interest), which were cloned either side of a kanamycin resistance cassette flanked by *loxP* sites. Arms A and B were generated by PCR and cloned into pCM184 (Marx and Lidstrom, 2002) using the primers and restriction sites shown in Supporting Information Table S3, followed by excision of the linear fragment by restriction digest, purification and transfer into the wild‐type or single‐deletion background strains by electroporation and selection on kanamycin‐containing plates as described (Crombie and Murrell, 2011). Removal of the kanamycin antibiotic cassette was accomplished by Cre recombinase, expressed from plasmid pCM157 (Marx and Lidstrom, 2002). Successful removal of the gene of interest was confirmed by PCR and sequencing using primers located outside the regions of homology (Supporting Information Table S3).

### Promoter reporter strains

Activity of the *mxaF*, *xoxF5* and *xoxF1* promoters was assayed by cloning the promoter fragments in front of a *gfp* gene in plasmid pMHA200 (Ali and Murrell, 2009). Upstream DNA (971 bp, 639 bp and 608 bp for *mxaF*, *xoxF5*, and *xoxF1* respectively) was amplified by PCR using the primers shown in Supporting Information Table S3. The resultant amplicons were cloned into pJet1.2 (Thermo Fisher Scientific, UK), digested with XbaI, blunted using T4 polymerase (Fermentas, Thermo Fisher Scientific), excised with KpnI, purified from a gel and ligated with pMHA200 treated similarly, except cut initially with EcoRI, resulting in plasmids pMHA_0471, pMHA_1587 and pMHA_3149 for *mxaF*, *xoxF5* and *xoxF1*, respectively. Integrity of these plasmids was checked by PCR, using primers pMHA_TF/pMHA_TR (Supporting Information Table S3) annealing to the pMHA200 backbone, and restriction digest. These constructs, together with the parental vector pMHA200, were transferred into all strains by electroporation, as previously described (Crombie and Murrell, 2011), and selected on kanamycin‐containing plates. Colonies were transferred to liquid medium containing kanamycin and grown to late exponential phase without Ln and then transferred to plastic universal tubes (5 ml culture volume) and grown with or without lanthanum (5 μM), in triplicate. Cells were harvested by centrifugation, resuspended in 400 μl phosphate buffer (pH 5.5, 20 mM) and 200 μl of the cells suspension was assayed for fluorescence in 96‐well black microplates using a Spectramax iD5 plate reader (Molecular Devices, San Jose, CA, USA) with excitation and emission at 485 and 535 nm respectively. The density of a 1/4 dilution was determined at 540 nm and fluorescence expressed as relative fluorescence units per unit culture density, subtracting the background values obtained with the promoter‐less vector.

### Sequencing of *mxaY*

DNA was extracted from the wild type and strain Δ*xoxF5* using standard methods (Sambrook *et al*., 2001). The entire *mxaY* gene (Msil_0484) was amplified by PCR using primers 484_F/484_R (Supporting Information Table S3) and sequenced using the same primers.

### Statistical analysis

Growth rate of cultures was determined during the exponential growth phase using a minimum of three data points. Since minor differences in culture medium or the condition (e.g. growth stage) of inoculating cultures can affect growth rates, statistical differences were only computed based on the growth of the same strain, in the presence or absence of Ln, that is, between cultures set up using the same parental culture, and not between strains. Comparisons between experimental conditions were carried out using Student's *t*‐test.

### Proteomic analysis

Wild‐type cells were grown in 25 ml of DNMS medium in 120 ml serum vials supplied with methanol (0.1% v/v) or ethanol (0.05% v/v) with or without lanthanum (5 μM) and harvested at late exponential phase. For mutant strains Δ*mxaF*Δ*xoxF5* and Δ*mxaF*Δ*xoxF1*, cells were harvested by pooling three replicates of each growth condition at the conclusion of the growth experiments described above. Cells were harvested by centrifugation (3500*g*, 20 min, 20°C), washed in lysis buffer (25 mM Tris (pH 7.5), 25 mM NaCl, protease inhibitor #2 (Merck 539132) (reconstituted as recommended and used at 1% v/v) and resuspended in 0.5 ml of the same buffer. Cells were broken by sonication (5 cycles of 15 s on and 20 s off) and centrifuged at 16 000*g*, 15 min, 4°C to remove cell debris. The extract was submitted to the Proteomics Facility, John Innes Centre (Norwich, UK) for analysis. The proteins were precipitated from the extract with five volumes of acetone (Nickerson and Doucette, 2020). The resulting pellets were dissolved in 100 μl of 2.5% sodium deoxycholate (SDC; Merck). Protein was quantified using the bicinchoninic acid assay (Thermo Fisher Scientific) and 100 μg of protein were reduced, alkylated, and digested with trypsin in the presence of 0.2 M EPPS buffer (Merck) and 2.5% SDC according to standard procedures. After the digest, the SDC was precipitated by adjusting to 0.2% trifluoroacetic acid (TFA), and the clear supernatant subjected to C18 solid‐phase extraction (SPE) (OMIX tips; Agilent). Tandem mass tag (TMT) labelling was performed using a TMT10plex™ kit (Lot TL274393, Thermo) according to the manufacturer's instructions with slight modification; the dried peptides were dissolved in 90 μl of 0.2 M EPPS buffer/10% acetonitrile, and 200 μg TMT reagent dissolved in 22 μl of acetonitrile was added. Aliquots of 2 μl from each sample were combined and analysed on the mass spectrometer (see below) to check labelling efficiency and estimate total sample abundances. The main sample aliquots were combined correspondingly and desalted using a C18 Sep‐pak cartridge (Waters). The eluted peptides were dissolved in 500 μl of 10 mM NH_4_HCO_3_ and fractionated by high pH reversed‐phase HPLC. Using an ACQUITY Arc Bio System (Waters), the samples were loaded to a Kinetex® 5 μm EVO C18 100 Å LC Column 250 × 4.6 mm (Phenomenex). Fractionation was performed with the following gradient of solvents A (water), B (acetonitrile), and C (25 mM NH_4_HCO_3_) at a flow rate of 1 ml min^−1^. Solvent C was kept at 10% throughout the gradient; solvent B: 0–5 min: 5%, 5–80 min: 5%–60%, 80–90 min: 60%–80%, followed by 5 min at 80% and re‐equilibration to 5%. Fractions of 1 ml were collected, dried down, and concatenated to produce 25 final fractions for MS analysis.

Aliquots were analysed by nanoLC‐MS/MS on an Orbitrap Eclipse™ Tribrid™ mass spectrometer coupled to an UltiMate® 3000 RSLCnano LC system (Thermo Fisher Scientific, Hemel Hempstead, UK). The samples were loaded onto a trap column (nanoEase M/Z Symmetry C18 Trap Column, Waters, Wilmslow, UK) with 0.1% TFA at 15 μl min^−1^ for 3 min. The trap column was then switched in‐line with the analytical column (nanoEase M/Z column, HSS C18 T3, 1.8 μm, 100 Å, 250 mm × 0.75 μm, Waters) for separation using the following gradient of solvents A (water, 0.1% formic acid) and B (80% acetonitrile, 0.1% formic acid) at a flow rate of 0.2 μl min^−1^: 0–3 min 3% B (parallel to trapping); 3–10 min linear increase B to 12%; 10–105 min increase B to 50%; followed by a ramp to 99% B and re‐equilibration to 3% B. Data were acquired with the following parameters in positive ion mode: MS1/OT: resolution 120 K, profile mode, mass range *m/z* 400–1800, automatic gain control (AGC) target 100%, max inject time 50 ms; MS2/IT: data dependent analysis with the following parameters: top10 in IT turbo mode, centroid mode, quadrupole isolation window 1.0 Da, charge states 2–5, threshold 1.9e^4^, collision energy (CE) = 33, AGC target 200%, max inject time 70 ms, dynamic exclusion 1 count/7 s/±7 ppm; MS3 synchronous precursor selection (SPS): 10 SPS precursors, isolation window 1 Da, higher‐energy C‐trap dissociation (HCD) fragmentation with CE = 65, Orbitrap Turbo TMT and TMTpro resolution 15 k, AGC target 200%, max inject time 105 ms, real‐time search: protein database *Methylocella sylvestris* (NCBI, GCA_000021745.1_ASM2174v1_translated_cds.faa, number of entries: 3821), 1 missed cleavage, oxidation (M) as variable, carbamidomethyl (C) and TMT6plex as fixed modifications, Xcorr = 1, dCn = 0.05.

The raw data were processed and quantified in Proteome Discoverer 2.4.1.15 (Thermo) using the incorporated search engine Sequest HT and the Mascot search engine (Matrix Science, London, UK; Mascot version 2.7.0). The processing workflow included recalibration of MS1 spectra (RC), precursor ion quantification by most confident centroid (20 ppm), fasta database *M. sylvestris* and common contaminants, precursor/fragment tolerance 6 ppm/0.6 Da, variable/fixed modifications were oxidation (M)/carbamidomethyl (C) and TMT6plex. The consensus workflow included the following parameters: unique peptides (protein groups), intensity‐based abundance, TMT channel correction values applied (TL274393), co‐isolation/SPS matches thresholds 50%/80%, normalization on total peptide abundances, protein abundance‐based ratio calculation, missing values imputation by low abundance resampling, hypothesis testing by *t*‐test (background based), adjusted *P*‐value calculation by BH method.

## Supporting information


**Table S1.** Abundance (% of total protein for each sample) of PQQ‐ADH in cell extract of *Methylocella silvestris* grown on methanol or ethanol in the absence or presence of lanthanum.
**Table S2.** Proteins differentially regulated in *M. silvestris* BL2 cells grown on A) methanol or B) ethanol, in response to lanthanum. The fold‐change (FC) of proteins upregulated in lanthanum‐containing medium is shown with a positive log_2_ (FC) value and downregulated proteins are shown with a negative value. Only proteins differentially expressed at |log_2_ (FC)| ≥ 2 and adjusted *p* value ≤0.01 are shown. Proteins regulated in both methanol‐ and ethanol‐grown cells are shown in bold type.
**Table S3.** Primer sequences.

**Table S4.** Table S4 is included as a separate Excel spreadsheet file.
**Fig. S1**. Detail of alignment of *M. silvestris* PQQ‐ADH sequences with other representative examples. The catalytic aspartate residue and calcium or lanthanide‐coordinating residue (A or D) (at positions 303 and 305 respectively in MxaF from *Methylorubrum extorquens*) are indicated with arrows.
**Fig. S2.** Growth curves of the wild type during growth on methanol (A) or ethanol (B). Error bars show the standard error of the mean (n = 3). Panel A shows a representative of three independent experiments.
**Fig. S3**. Double mutant strain *ΔmxaFΔxoxF5* (A) or the wild type (B) were grown on methanol (0.1% v/v) in the presence (red) or absence (black) of 4% v/v acetylene in the vial headspace. Error bars show the standard error of the mean (n = 3).
