# Supplementary material for: The effect of lanthanum on growth and gene expression in a facultative methanotroph
Source: Environ Microbiol. 2021 Aug 12;24(2):596–613. doi: 10.1111/1462-2920.15685 (PMC9291206; doi:10.1111/1462-2920.15685)
Supplement: Supplementary file 1 — Table S1. Abundance (% of total protein for each sample) of PQQ‐ADH in cell extract of Methylocella silvestris grown on methanol or ethanol in the absence or presence of lanthanum. Table S2. Proteins differentially regulated in M. silvestris BL2 cells grown on A) methanol or B) ethanol, in response to lanthanum. The fold‐change (FC) of proteins upregulated in lanthanum‐containing medium is shown with a positive log2 (FC) value and downregulated proteins are shown with a negative value. Only proteins differentially expressed at |log2 (FC)| ≥ 2 and adjusted p value ≤0.01 are shown. Proteins regulated in both methanol‐ and ethanol‐grown cells are shown in bold type. Table S3. Primer sequences. [file EMI-24-596-s002.docx]

**Supplementary Information**

**Table S1.** Abundance (% of total protein for each sample) of PQQ-ADH in cell extract of *Methylocella silvestris* grown on methanol or ethanol in the absence or presence of lanthanum.

| Strain | | Wild type | | | | Δ*mxaF*Δ*xoxF5* | | | | Δ*mxaF*Δ*xoxF1* | | |
| --- | --- | --- | --- | --- | --- | --- | --- | --- | --- | --- | --- | --- |
| substrate | | Methanol | | Ethanol | | Methanol | | Ethanol | | Methanol | | Ethanol |
| Locus tag | Protein | **-La** | +La | -La | +La | -La | +La | -La | +La | -La | -La | |
| Msil_0471 | MxaF | 9.7 | 1.06 | 12.0 | 1.9 | G.K. | G.K. | G.K. | G.K. | G.K. | G.K. | |
| Msil_1587 | XoxF5 | 0.082 | 4.45 | 0.012 | 11.0 | G.K. | G.K. | G.K. | G.K. | 0.237 | 0.021 | |
| Msil_2260 | XoxF5(2) | N.D. | N.D. | N.D. | N.D. | N.D. | N.D. | N.D. | N.D. | N.D. | N.D. | |
| Msil_2867 | Grp 9 ADH | 0.014 | 0.012 | 0.019 | 0.006 | 0.032 | 0.033 | 0.024 | 0.028 | 0.029 | 0.021 | |
| Msil_3149 | XoxF1 | 0.0076 | 0.0010 | 0.0114 | 0.0012 | 0.0221 | 0.0003 | 0.0080 | 0.0003 | G.K. | G.K. | |
| Msil_3387 | XoxF3 | 0.00003 | 0.00003 | 0.00002 | 0.00002 | 0.00017 | 0.00016 | 0.00006 | 0.00007 | 0.00012 | 0.00006 | |
| Msil_3733 | Grp 9 ADH | 0.004 | 0.008 | 0.006 | 0.029 | 0.004 | 0.005 | 0.003 | 0.006 | 0.007 | 0.004 | |

G.K., gene knockout in this strain; N.D., not detected.

**Table S2.** Proteins differentially regulated in *M. silvestris* BL2 cells grown on A) methanol or B) ethanol, in response to lanthanum. The fold-change (FC) of proteins upregulated in lanthanum-containing medium is shown with a positive log_2_ (FC) value and downregulated proteins are shown with a negative value. Only proteins differentially expressed at |log_2_ (FC)| ≥ 2 and adjusted *p* value ≤ 0.01 are shown. Proteins regulated in both methanol- and ethanol-grown cells are shown in bold type.

| 1. **Methanol** |  |  |  |
| --- | --- | --- | --- |
| locus_tag | Description | log(2) FC | Adj. *p*-value |
| **Msil_0271** | **LanM EF-hand-containing_protein** | **5.3** | **2.3E-15** |
| Msil_0461 | fumarate_lyase | 2.5 | 3.9E-07 |
| **Msil_0471** | **MxaF PQQ-dependent_dehydrogenase** | **-3.2** | **2.8E-12** |
| **Msil_0472** | **MxaJ extracellular_solute-binding_protein** | **-4.8** | **2.3E-15** |
| **Msil_0473** | **MxaG cytochrome_c_class_I** | **-3.9** | **2.3E-15** |
| Msil_0474 | MxaI methanol_dehydrogenase_beta_subunit | -2.8 | 1.3E-09 |
| **Msil_0475** | **MxaR** | **-3.1** | **5.2E-12** |
| **Msil_0476** | **MxaS conserved_hypothetical_protein** | **-3.4** | **4.8E-14** |
| Msil_0477 | MxaA_protein,_putative | -2.1 | 1.5E-05 |
| Msil_0478 | MxaC von_Willebrand_factor_type_A | -2.2 | 4.1E-06 |
| Msil_0479 | MxaK conserved_hypothetical_protein | -2.5 | 8.8E-08 |
| Msil_0480 | MxaL von_Willebrand_factor_type_A | -2.6 | 3.9E-08 |
| Msil_0481 | MxaD_protein,_putative | -3.3 | 9.3E-13 |
| **Msil_0482** | **MxaE 40-residue_YVTN_family_protein** | **-2.4** | **7.6E-07** |
| **Msil_0483** | **MxaH conserved_hypothetical_protein** | **-3.2** | **4.3E-12** |
| **Msil_0485** | **MxaB two_component_transcriptional_regulator** | **-4.1** | **2.3E-15** |
| Msil_0486 | diguanylate_cyclase | -2.2 | 6.4E-05 |
| **Msil_0535** | **RNA_chaperone_Hfq** | **-2.1** | **1.1E-05** |
| Msil_1509 | nitrate_reductase,_beta_subunit | 2.7 | 2.5E-08 |
| **Msil_1510** | **nitrate_reductase,_alpha_subunit** | **2.6** | **2.9E-07** |
| Msil_1512 | nitrite_transporter | 2.4 | 2.5E-06 |
| **Msil_1585** | **XoxJ extracellular_solute-binding_protein** | **2.5** | **5.6E-07** |
| **Msil_1586** | **XoxG putative_cytochrome_c_protein** | **3.1** | **5.0E-11** |
| **Msil_1587** | **XoxF5 PQQ-dependent_dehydrogenase** | **5.8** | **2.3E-15** |
| Msil_1867 | conserved_hypothetical_protein | -2.2 | 5.8E-06 |
| Msil_2495 | Rieske_(2Fe-2S)_domain_protein | -2.4 | 8.5E-06 |
| Msil_2592 | bacterioferritin | -2.0 | 5.4E-05 |
| Msil_2868 | conserved_hypothetical_protein | -2.2 | 4.1E-06 |
| **Msil_3146** | **ABC-transporter** | **-2.2** | **4.0E-06** |
| **Msil_3147** | **XoxG cytochrome_c_class_I** | **-2.7** | **4.3E-09** |
| **Msil_3148** | **XoxJ extracellular_solute-binding_protein** | **-6.0** | **2.3E-15** |
| **Msil_3149** | **XoxF1 PQQ-dependent_dehydrogenase** | **-2.9** | **5.1E-10** |
| Msil_3615 | Electron_transfer_flavoprotein_alpha_subunit | -6.6 | 2.3E-15 |
| Msil_3626 | nitrogen_fixation_protein | -4.0 | 2.3E-15 |
| Msil_3627 | nitrogen_fixation_protein_NifX | -3.9 | 2.3E-15 |
| Msil_3632 | nitrogenase_iron_protein | -2.4 | 5.3E-07 |
| Msil_3633 | response_regulator_receiver_protein | -2.2 | 9.4E-06 |
| Msil_3634 | conserved_hypothetical_protein | -2.0 | 5.4E-05 |
| Msil_3636 | ferredoxin | -2.2 | 7.1E-05 |
| Msil_3644 | HesB/YadR/YfhF-family_protein | -4.2 | 2.3E-15 |
| Msil_3645 | 4Fe-4S_ferredoxin_iron-sulfur_binding_domain_protein | -3.6 | 2.3E-15 |
| Msil_3649 | conserved_hypothetical_protein | -2.0 | 4.3E-05 |
| Msil_3676 | conserved_hypothetical_protein | 2.1 | 9.9E-05 |
| Msil_3840 | Alcohol_dehydrogenase_zinc-binding_domain_protein | -2.3 | 1.4E-06 |
|  |  |  |  |
| 1. **Ethanol** |  |  |  |
| locus_tag | Description | log(2) FC | Adj. p-value |
| Msil_0111 | major_facilitator_superfamily_MFS_1 | 2.2 | 4.2E-03 |
| **Msil_0271** | **LanM EF-hand-containing_protein** | **4.7** | **4.1E-13** |
| Msil_0407 | ABC_transporter_domain_protein | 2.6 | 7.2E-04 |
| **Msil_0471** | **MxaF PQQ-dependent_dehydrogenase** | **-2.6** | **1.2E-03** |
| **Msil_0472** | **MxaJ extracellular_solute-binding_protein** | **-4.2** | **8.6E-10** |
| **Msil_0473** | **MxaG cytochrome_c_class_I** | **-2.9** | **2.6E-04** |
| **Msil_0475** | **MxaR** | **-2.5** | **2.2E-03** |
| **Msil_0476** | **MxaS conserved_hypothetical_protein** | **-2.4** | **4.1E-03** |
| **Msil_0482** | **MxaE 40-residue_YVTN_family_protein** | **-2.9** | **2.8E-04** |
| **Msil_0483** | **MxaH conserved_hypothetical_protein** | **-2.7** | **7.6E-04** |
| **Msil_0485** | **MxaB two_component_transcriptional_regulator** | **-3.4** | **6.2E-06** |
| **Msil_0535** | **RNA_chaperone_Hfq** | **-5.1** | **7.0E-15** |
| Msil_0657 | V-type_H(+)-translocating_pyrophosphatase | 2.8 | 1.4E-04 |
| Msil_0734 | porin | 2.3 | 2.8E-03 |
| Msil_0749 | Phasin_protein. | -3.4 | 4.3E-06 |
| Msil_0764 | aldo/keto_reductase | -2.4 | 5.5E-03 |
| Msil_1214 | Glycine_dehydrogenase_(decarboxylating) | -2.4 | 5.6E-03 |
| Msil_1504 | Predicted_acetyltransferase | -2.5 | 2.4E-03 |
| Msil_1508 | nitrate_reductase_molybdenum_cofactor_assembly_chaperone | -4.8 | 4.1E-13 |
| **Msil_1510** | **nitrate_reductase,_alpha_subunit** | **-2.4** | **4.5E-03** |
| **Msil_1585** | **XoxJ extracellular_solute-binding_protein** | **3.4** | **1.5E-06** |
| **Msil_1586** | **XoxG putative_cytochrome_c_protein** | **4.2** | **3.1E-10** |
| **Msil_1587** | **XoxF5 PQQ-dependent_dehydrogenase** | **9.8** | **7.0E-15** |
| Msil_1649 | methane/phenol/toluene_hydroxylase | -2.5 | 2.9E-03 |
| Msil_1651 | Propane_monooxygenase | -3.1 | 4.1E-05 |
| Msil_1709 | hypothetical_protein | -4.0 | 8.5E-07 |
| Msil_1716 | succinyl-CoA_synthetase,_beta_subunit | 2.6 | 6.5E-04 |
| Msil_1837 | conserved_hypothetical_protein | -2.9 | 6.5E-04 |
| Msil_1860 | ammonium_transporter | 2.5 | 7.2E-04 |
| Msil_2256 | major_facilitator_superfamily_MFS_1 | 2.3 | 7.2E-03 |
| Msil_2414 | ethanolamine_transproter | 2.5 | 1.3E-03 |
| Msil_2419 | Serine_O-acetyltransferase | -2.5 | 2.2E-03 |
| Msil_2623 | SSS_sodium_solute_transporter_superfamily | 2.4 | 2.2E-03 |
| Msil_2659 | protein_of_unknown_function_DUF1326 | 2.2 | 5.2E-03 |
| Msil_3046 | conserved_hypothetical_protein | 2.6 | 6.1E-04 |
| Msil_3051 | conserved_hypothetical_protein | 2.9 | 1.1E-04 |
| Msil_3059 | hypothetical_protein | 2.8 | 2.3E-04 |
| Msil_3064 | hypothetical_protein | 3.2 | 8.3E-06 |
| Msil_3065 | hypothetical_protein | 2.7 | 2.5E-04 |
| **Msil_3146** | **ABC-transporter** | **-2.8** | **5.0E-04** |
| **Msil_3147** | **XoxG cytochrome_c_class_I** | **-2.5** | **3.0E-03** |
| **Msil_3148** | **XoxJ extracellular_solute-binding_protein** | **-4.5** | **4.8E-11** |
| **Msil_3149** | **XoxF1 PQQ-dependent_dehydrogenase** | **-3.3** | **1.1E-05** |
| Msil_3260 | precorrin-4_C11-methyltransferase | -2.5 | 2.5E-03 |
| Msil_3602 | conserved_hypothetical_protein,_proteobacterial | 2.6 | 6.8E-04 |
| Msil_3696 | UDP-glucose_4-epimerase | -2.8 | 3.4E-04 |
| Msil_3733 | Pyrrolo-quinoline_quinone | 2.3 | 3.8E-03 |
| Msil_3774 | nitrite_reductase_(NAD(P)H),_large_subunit | 3.6 | 1.3E-07 |
| Msil_3775 | Rieske_(2Fe-2S)_domain_protein | 2.8 | 1.5E-04 |

**Table S3.** Primer sequences

| Name | Sequence (5′ - 3′) | | | Target | | | | Description | | | REN | | | |  |  |  |
| --- | --- | --- | --- | --- | --- | --- | --- | --- | --- | --- | --- | --- | --- | --- | --- | --- | --- |
| **qPCR assays** | | | | | | | | | | | | | | |  |  |  |
| 0471_qF | | CACGCTCGTGGCCATTTCTC | *mxaF* | | | | qPCR_forward | | |  | | | |  |  |  |  |
| 0471_qR | | CCCGGCATCACCCAATTGTC | *mxaF* | | | | qPCR_reverse | | |  | | | |  |  |  |  |
| 1587_qF | | GATTGCCGGAGCCGTAGTAG | *xoxF5* | | | | qPCR_forward | | |  | | | |  |  |  |  |
| 1587_qR | | ACCTGGGAAGGCGATCAGTG | *xoxF5* | | | | qPCR_reverse | | |  | | | |  |  |  |  |
| 2260_qF | | GCGCATAGGCGATGTGAAAC | *xoxF5(2)* | | | | qPCR_forward | | |  | | | |  |  |  |  |
| 2260_qR | | CAATCCCGCGGCCTATTCAG | *xoxF5(2)* | | | | qPCR_reverse | | |  | | | |  |  |  |  |
| 3149_qF2 | | CCGGCACTCGGAACGTATAG | *xoxF1* | | | | qPCR_forward | | |  | | | |  |  |  |  |
| 3149_qR2 | | GCCTCGATCTCGAAGACAAG | *xoxF1* | | | | qPCR_reverse | | |  | | | |  |  |  |  |
| 3387_qF | | CCGGAACCCGTCTTGAACTG | *xoxF3* | | | | qPCR_forward | | |  | | | |  |  |  |  |
| 3387_qR | | TCTGGAGCGGAACCGTAGTC | *xoxF3* | | | | qPCR_reverse | | |  | | | |  |  |  |  |
| mmoX_qF | | CGCTGAGGGTGCATGAGTTC | *mmoX* | | | | qPCR_forward | | |  | | | |  |  |  |  |
| mmoX_qR | | AGACGCTCTGGCACTCGTAG | *mmoX* | | | | qPCR_reverse | | |  | | | |  |  |  |  |
| rpoB_qF | | AGGTCACGTTGCGCCTCATC | *rpoB* | | | | qPCR_forward | | |  | | | |  |  |  |  |
| rpoB_qR | | GCGGCATGTCGCCCATATAG | *rpoB* | | | | qPCR_reverse | | |  | | | |  |  |  |  |
| **Gene knockouts** | | | | | | | | | | | | | | | | |  |
| 0471_AF | | ATCAAGATCTGGGCGTTCCGGGTAGGAGAC | *mxaF* | | | Homology-A, forward | | | | | | BglII | | | |  |  |
| 0471_AR | | ATCAGGTACCCTCACGTTCCTGCCCAGTAG | *mxaF* | | | Homology-A, reverse | | | | | | KpnI | | | |  |  |
| 0471_BF | | ATCAACGCGTATCGACGCCCTCGAATTGAC | *mxaF* | | | Homology-B, forward | | | | | | MluI | | | |  |  |
| 0471_BR | | ATCAGAGCTCCATAGGCGGAGCGATAATAG | *mxaF* | | | Homology-B, reverse | | | | | | SacI | | | |  |  |
| 1587_AF | | ATCAAGATCTGCGCCCACGTAGATAAGC | *xoxF5* | | | Homology-A, forward | | | | | | BglII | | | |  |  |
| 1587_AR | | TATCGGTACCTAAGCCTGCCCACTTAATCG | *xoxF5* | | | Homology-A, reverse | | | | | | KpnI | | | |  |  |
| 1587_BF | | ATCAACGCGTGGCAGCGCTTCTTGTATGTC | *xoxF5* | | | Homology-B, forward | | | | | | MluI | | | |  |  |
| 1587_BR | | TATCGAGCTCTGAGGTCCGCTGCTTCTAAC | *xoxF5* | | | Homology-B, reverse | | | | | | SacI | | | |  |  |
| 3149_AF | | ATCAAGATCTCCTTGCGCGAGGCGAAGAC | *xoxF1* | | | Homology-A, forward | | | | | | BglII | | | |  |  |
| 3149_AR | | TATCGGTACCCGGCGCCCTCTATACGTTCC | *xoxF1* | | | Homology-A, reverse | | | | | | KpnI | | | |  |  |
| 3149_BF | | ATCAACGCGTCTTGCCATCGGCGTAGTTCAC | *xoxF1* | | | Homology-B, forward | | | | | | MluI | | | |  |  |
| 3149_BR | | TTAAACCGGTAGGGCAGCAAAGCGAGAG | *xoxF1* | | | Homology-B, reverse | | | | | | AgeI | | | |  |  |
| **Verification** | | | | | | | | | | | | | | | | |  |
| 0471_TF | | TTACGAGTCGCACAGATCAG | *mxaF* | | | | Forward | | |  | | | |  |  |  |  |
| 0471_TR | | CAATTCGCGGATCTTTCCAG | *mxaF* | | | | Reverse | | |  | | | |  |  |  |  |
| 1587_TF | | AGTCCCAAGCAGGCCTTCTC | *xoxF5* | | | | Forward | | |  | | | |  |  |  |  |
| 1587_TR | | CCAGATCGGCACGATGTTTC | *xoxF5* | | | | Reverse | | |  | | | |  |  |  |  |
| 3149_TF | | ATGATCGGCGAGCGACTGAC | *xoxF1* | | | | Forward | | |  | | | |  |  |  |  |
| 3149_TR | | AACCGGCTCTATCGCCCTTG | *xoxF1* | | | | Reverse | | |  | | | |  |  |  |  |
| pMHA_TF | | GTGCCCATTAACATCACC | pMHA200 | | | | Forward | | |  | | | |  |  |  |  |
| pMHA_TR | | AAGCAGAAGGCCATCCTGAC | pMHA200 | | | | Reverse | | |  | | | |  |  |  |  |
| 484_F | | GCGCCATAGAACGTCTAAGG | *mxaY* | | | | Forward | | |  | | | |  |  |  |  |
| 484_R | | GCGCGTCTGCTTTCATAGTG | *mxaY* | | | | Reverse | | |  | | | |  |  |  |  |
| **MDH promoters** | | | | | | | | | | | | | | | | |  |
| 0471_PrF | | ATCAGGTACCACGTTCCTGCCCAGTAGAGAC | | | P_mxaF_ | | | | Forward | | | | KpnI | | | | |
| 0471_PrR | | ATCATCTAGACGCGCCTTAGTTCTGAACGTGC | | | P_mxaF_ | | | | Reverse | | | | XbaI | | | | |
| 1587_PrF | | ATATGGTACCGACCGTATGTATGCGGACTG | | | P_xoxF5_ | | | | Forward | | | | KpnI | | | | |
| 1587_PrR | | ATCATCTAGACAACAGCCCGCAGGCTCAG | | | P_xoxF5_ | | | | Reverse | | | | XbaI | | | | |
| 3149_PrF | | ATCAGGTACCAGGCGTCACGAAATCATCGGCATTG | | | P_xoxF1_ | | | | Forward | | | | KpnI | | | | |
| 3149_PrR | | ATCATCTAGAGCGACAATGAACGACCTAGC | | | P_xoxF1_ | | | | Reverse | | | | XbaI | | | | |

**Table S4**. Table S4 is included as a separate Excel spreadsheet file, Supplementary Table S4.xlsx.


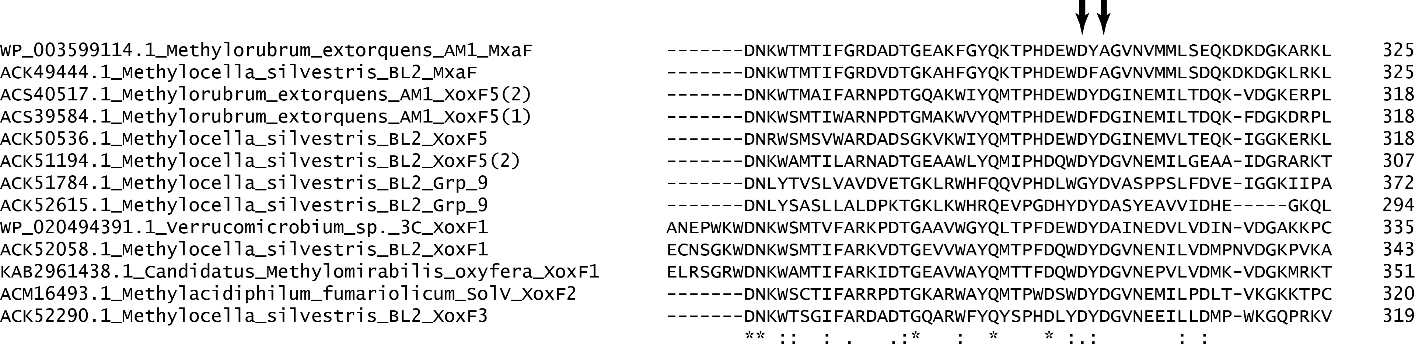


**Figure S1**. Detail of alignment of *M. silvestris* PQQ-ADH sequences with other representative examples*.* The catalytic aspartate residue and calcium or lanthanide-coordinating residue (A or D) (at positions 303 and 305 respectively in MxaF from *Methylorubrum extorquens*) are indicated with arrows.


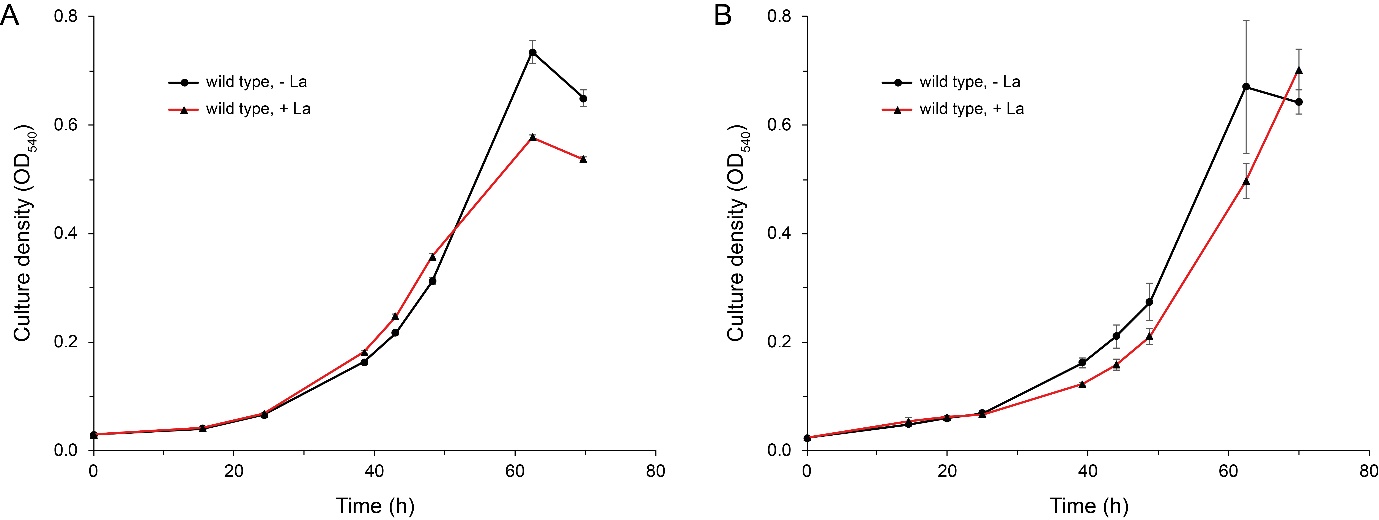


**Figure S2.** Growth curves of the wild type during growth on methanol (A) or ethanol (B). Error bars show the standard error of the mean (n=3). Panel A shows a representative of three independent experiments.


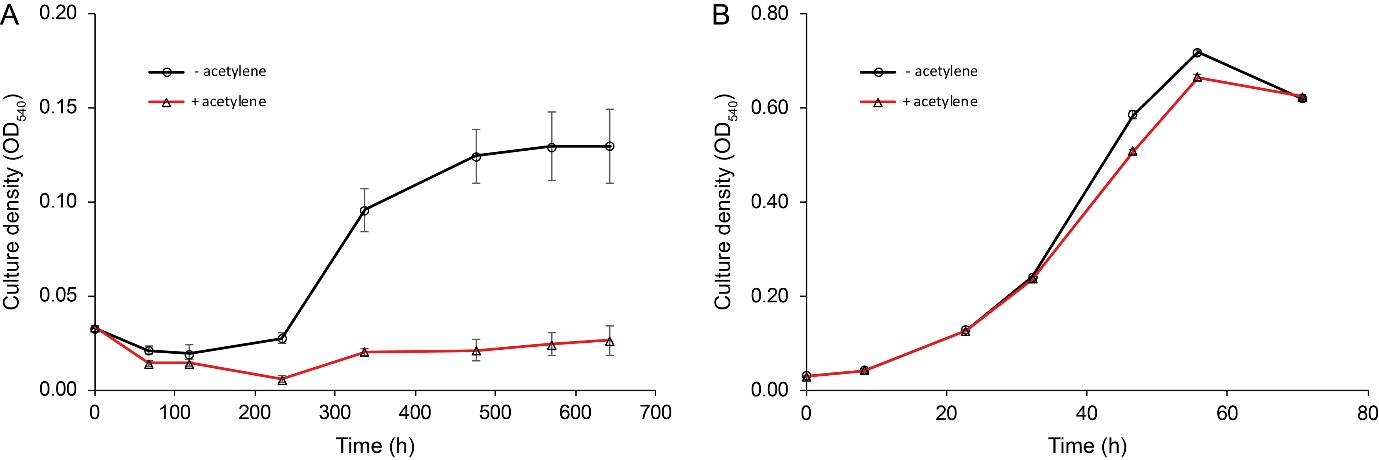


**Figure S3**. Double mutant strain Δ*mxaF*Δ*xoxF5* (A) or the wild type (B) were grown on methanol (0.1% v/v) in the presence (red) or absence (black) of 4% v/v acetylene in the vial headspace. Error bars show the standard error of the mean (n=3).
